# Estradiol Activates β-Catenin Dependent Transcription in Neurons

**DOI:** 10.1371/journal.pone.0005153

**Published:** 2009-04-10

**Authors:** Olga Varea, Juan Jose Garrido, Ana Dopazo, Pablo Mendez, Luis Miguel Garcia-Segura, Francisco Wandosell

**Affiliations:** 1 Centro de Investigación Biomédica en Red sobre Enfermedades Neurodegenerativas (CIBERNED) and Centro de Biología Molecular “Severo Ochoa”, CSIC-UAM, Madrid, Spain; 2 Genomics Unit, Centro Nacional de Investigaciones Cardiovasculares (CNIC), Madrid, Spain; 3 Laboratory of Neuroactive Steroids, Instituto Cajal, CSIC, Madrid, Spain; 4 Laboratory of Neuronal Polarity, Instituto Cajal, CSIC, Madrid, Spain; Medical College of Georgia, United States of America

## Abstract

Estradiol may fulfill a plethora of functions in neurons, in which much of its activity is associated with its capacity to directly bind and dimerize estrogen receptors. This hormone-protein complex can either bind directly to estrogen response elements (ERE's) in gene promoters, or it may act as a cofactor at non-ERE sites interacting with other DNA-binding elements such as AP-1 or c-Jun. Many of the neuroprotective effects described for estrogen have been associated with this mode of action. However, recent evidence suggests that in addition to these “genomic effects”, estrogen may also act as a more general “trophic factor” triggering cytoplasmic signals and extending the potential activity of this hormone. We demonstrated that estrogen receptor alpha associates with β-catenin and glycogen synthase kinase 3 in the brain and in neurons, which has since been confirmed by others. Here, we show that the action of estradiol activates β-catenin transcription in neuroblastoma cells and in primary cortical neurons. This activation is time and concentration-dependent, and it may be abolished by the estrogen receptor antagonist ICI 182780. The transcriptional activation of β-catenin is dependent on lymphoid enhancer binding factor-1 (LEF-1) and a truncated-mutant of LEF-1 almost completely blocks estradiol TCF-mediated transcription. Transcription of a TCF-reporter in a transgenic mouse model is enhanced by estradiol in a similar fashion to that produced by Wnt3a. In addition, activation of a luciferase reporter driven by the *engrailed* promoter with three LEF-1 repeats was mediated by estradiol. We established a cell line that constitutively expresses a dominant-negative LEF-1 and it was used in a gene expression microarray analysis. In this way, genes that respond to estradiol or Wnt3a, sensitive to LEF-1, could be identified and validated. Together, these data demonstrate the existence of a new signaling pathway controlled by estradiol in neurons. This pathway shares some elements of the insulin-like growth factor-1/Insulin and Wnt signaling pathways, however, our data strongly suggest that it is different from that of both these ligands. These findings may reveal a set of new physiological roles for estrogens, at least in the Central Nervous System (CNS).

## Introduction

Estrogens fulfill a wide range of functions during development and differentiation in mammals of both sexes. In addition to these functions, they are also thought to play an important role in neuroprotection [1]–[3]. The actions of estrogens have been classified as either “genomic actions” or “non-genomic, rapid actions”. The genomic actions are based on the capacity of the estrogen receptors (ERs) to bind to co-activators or co-repressors in order to enhance or inhibit the transcription of target genes, and it has been reported in many cell types (reviewed in [4]). This activity involves the dimerization of two receptor molecules mediated by the presence of the hormone and the generation of a macromolecular complex with co-regulators (reviewed in [5]). The ERs belong to the nuclear receptor superfamily and two receptors, alpha and beta, have been identified (NR3A1 and NR3A2, according to the nomenclature of the NRN Committee [6]. The structure of both receptors is similar containing a highly homologous DNA-binding region (95%) and a hormone binding region with weaker homology (69%), whereas the carboxy and amino-terminal regions are the most divergent regions (58% homology, reviewed in [7]). Apart from this genomic action, estrogen can trigger rapid “non-genomic signaling” associated with the activation of second messengers. Among these, the activation of the mitogen activated protein kinase (MAPK) [8], protein kinase C (PKC) [9] and phosphoinositide 3-kinase (PI3K) [10] signaling pathways has been described. Indeed, cooperation with insulin-like growth factor-1 (IGF-1) has been demonstrated and ERα has been reported to associate with p85, the regulatory subunit of PI3K [11]–[12]. Furthermore, estrogens may also act in a ligand-independent manner [13] and they may exert certain antioxidant effects that are independent of their receptors.

We recently demonstrated that ERα is linked to PI3K associated “cytoplasmic signaling” in the brain and in primary neurons, where estradiol can induce the immediate activation of Akt/PKB and the subsequent inhibition of glycogen synthase kinase 3 (GSK3). On the light of the important role proposed for GSK3 in neuronal survival [14] and in neuropathologies such as amyloid neurotoxicity [15], the role of this new estradiol signaling pathway merits further analysis.

We also identified novel complexes in which ERα, GSK3 and β-catenin were associated, demonstrating that the action of estradiol transiently stabilized β-catenin, which is disassociated from the complex with ERα [12]. The stabilization of β-catenin has been related with different functions of this protein. In addition to act in cell-cell adhesion, β-catenin also serves as a co-transcriptional regulator, modulating the functions of the T cell factor (TCF)/ lymphoid enhancer binding factor (LEF) proteins that are closely associated with Wnt signaling (reviewed in [16]–[19]). Hence, the stabilization of β-catenin by estradiol may also have effects on gene transcription.

In this study we have assessed whether the β-catenin, stabilized by estradiol in neurons, may exert a significant effect at the transcriptional level. We show that the stabilization of β-catenin by estradiol is correlated with the inhibition of GSK3 in neuronal-like cells (N2a-m), as well as in cortical neurons. Indeed, estradiol increased TCF/LEF-1 transcription in a dose-dependent manner. This increase in transcription was partially prevented by the addition of an estradiol antagonist and was mimicked by an alpha and beta specific-agonist. In neuronal cells, estradiol induced the formation of a TCF-DNA complex that was impaired by the presence of antibodies against LEF-1. Moreover, in cortical neurons from a TCF/LEF-1–βgal transgenic mouse, β-galactosidase activity was up-regulated by the action of estradiol. Finally, in a LEF-1 mutant cell line, we identified some genes that were differentially regulated by estradiol.

Together, all these data demonstrate that in addition to its nuclear action mediated through the ER, estradiol triggers a signal that recruits β-catenin and LEF-1 and that may be responsible for more wide-ranging actions of this hormone, at least in neurons.

## Results

### Estradiol transiently regulates the phosphorylation of GSK3 and the amount of β-catenin in N2a-m neuronal cells and cortical neurons

We recently demonstrated that estradiol inhibit GSK3 and stabilize β-catenin in the hippocampus and in hippocampal neurons. To further analyze this phenomenon, it was necessary to transfect neuronal cells. We first examined whether neuroblastoma cells behave like primary neurons concerning this estrogenic action. We found that **N2a-m** cells, a clone derived from NB2a (ATCC: CCL 131) and that has been maintained for several years in our laboratory, responded to estradiol in a similar manner as primary neurons, as demonstrated below.

**Figure 1:**
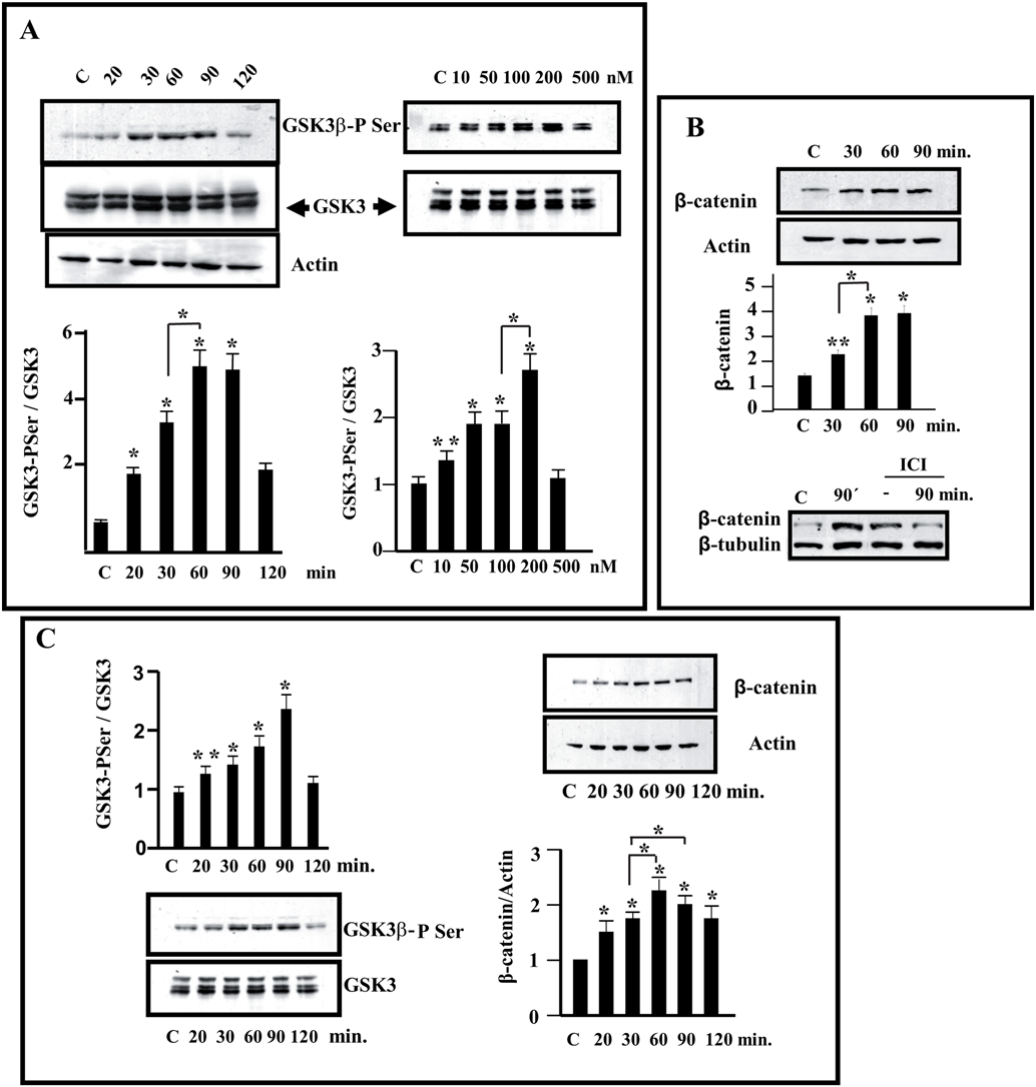
Treatment of estradiol increases Ser^9,21^-GSK3 phosphorylation and β-catenin accumulation in neuronal cells. (A)-Neuroblastoma N2a-m were treated with different doses of estradiol and for different times (20–120 min with 10–500 nM) and the maximum increase in GSK3β-PSer^9^ was observed when they were treated for 60 min with 100–200 nM estradiol. (B)- Stabilization of β-catenin. N2a-m cells treated with estradiol showed a clear increase in β-catenin that could be prevented by prior exposure to ICI 182780 (used 100× concentrated in comparison to estradiol), 60 minutes before estradiol treatment (lower panel). (C)-Cortical Primary Neurons (2DIV) were treated with estradiol (100 nM) and the maximum increase in GSK3β-PSer^9^ and the subsequent stabilization of β-catenin was clearly detected 60–90 min after the onset of exposure. In all cases diagram shows the mean normalized densitometry values and the corresponding standard deviations from at least three independent experiments. Asterisks indicate statistical significance (Student's t-test) ** (P≤0.05), * (P≤0.01). The single * or ** compares data to control whereas the bar between different points shows statistical differences between experimental values.

The effect of estradiol on this cell line was examined and we initially determined whether N2a-m cells expressed both α and β estrogen receptors when maintained in serum–free medium (Supplementary Figure S1). When N2a-m cells were exposed to estradiol, ERα-immunoreactivity became more concentrated in the nucleus in a time and dose dependent manner (Supplementary Figure S1). Thus, since these cells respond to estradiol and can be efficiently transfected, we analyzed whether exposure to estradiol could modify GSK3β-PSer using primary cortical neurons as a reference in parallel. Immunoreactivity against GSK3β-PSer was increased in N2a-m cells exposed to estradiol, reaching a maximum (5±1 fold, n = 4) 60–90 minutes after the addition of the hormone (Figure 1A). A similar time-course of GSK3β-PSer immunoreactivity was observed in cortical primary neurons (Figure 1C), reaching a maximum increase (2.2±0.5 fold; n = 3) 90 minutes after exposure to the hormone. The increase in serine phosphorylation of GSK3β was dose-dependent and was maximal in the range of 100 to 200 nM (2.5±0.5 fold; n = 3, Figure 1A).

The second important element in our analysis is β-catenin, as demonstrated in hippocampal neurons [12]. Thus, we determined whether inhibition of GSK3 was correlated with the stabilization of β-catenin in cell extracts from N2a-m and cortical neurons treated with estradiol (100 nM). The total amount of β-catenin increased in both N2a-m cells (3.5±0.5 fold; n = 3) (Figure 1A) and cortical neurons (2.2±0.3 folds, n = 3) (Figure 1C), with a time-course similar to that of the changes in GSK3-PSer (Figure 1 A and B). The increase in the stabilization of β-catenin by estradiol was prevented by ICI 182780, a specific ER receptor antagonist (*ICI/estradiol ratio was maintained 100×*) (Figure 1B).

**Figure 2:**
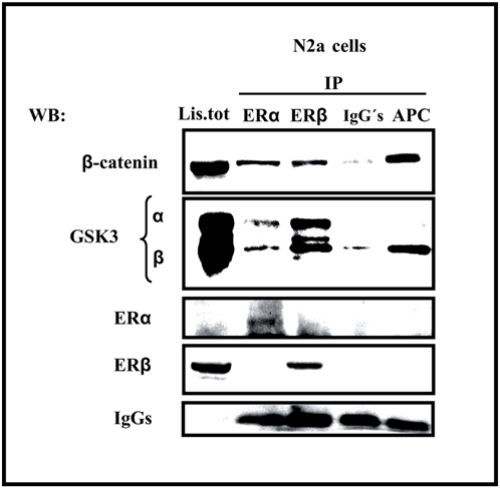
Estrogen receptors form a complex with β-catenin and GSK3 in N2a-m cells. ERα and ERβ were immunoprecipitated from N2a-m cells, and similar endogenous protein complexes were observed with both antibodies. Immunoprecipitation with an anti-APC antibody is shown as an internal control of a β-catenin interacting protein. Both GSK3 isoforms were detected in the immunocomplex recovered with ERα or ERβ antibodies, whereas GSK3β is the major isoform recovered with APC antibodies, used as a positive control. The immunoprecipitation also shows a fraction of β-catenin associated with both ER isoforms. IgG's represent the IP using irrelevant IgGs as a negative control.

A complex of ER α and β-catenin was recently detected in the hippocampus of female ovariectomized rats [12]. We assessed whether a similar complex was present in extracts from N2a-m cells. Our data showed that β-catenin was detected in complexes immunoprecipitated with antibodies against ERα or ERβ (Figure 2). Similarly, both GSK3α and β were detected in these complexes, although there was less GSK3 recovered when antibodies against ERα were used. As a positive control in these experiments, material was immunopreciptated with an antibody against Adenomatous Polyposis Coli protein (APC) and in these latter experiments, mostly GSK3β was detected (Figure 2).

### Estradiol increase the association of β-catenin to the neuronal membrane fraction

Given that β-catenin may fulfill two distinct cellular functions, as an adhesion molecule or as co-transcription factor. Two different pools have been described associated with different functions [16], [20]. Thus, we determined whether estradiol-mediated stabilization augmented either of the putative β-catenin pools. Membrane and nuclear fractions were obtained from cultured neurons exposed to the hormone for 30 and 60 minutes. Interestingly, the amount of β-catenin detected in the membrane fraction was increased at 30 and 60 minutes after exposure to estradiol whereas the nuclear fraction was almost unaffected (Supplementary Figure S2).

### Estradiol activates TCF-mediated transcription

**Figure 3:**
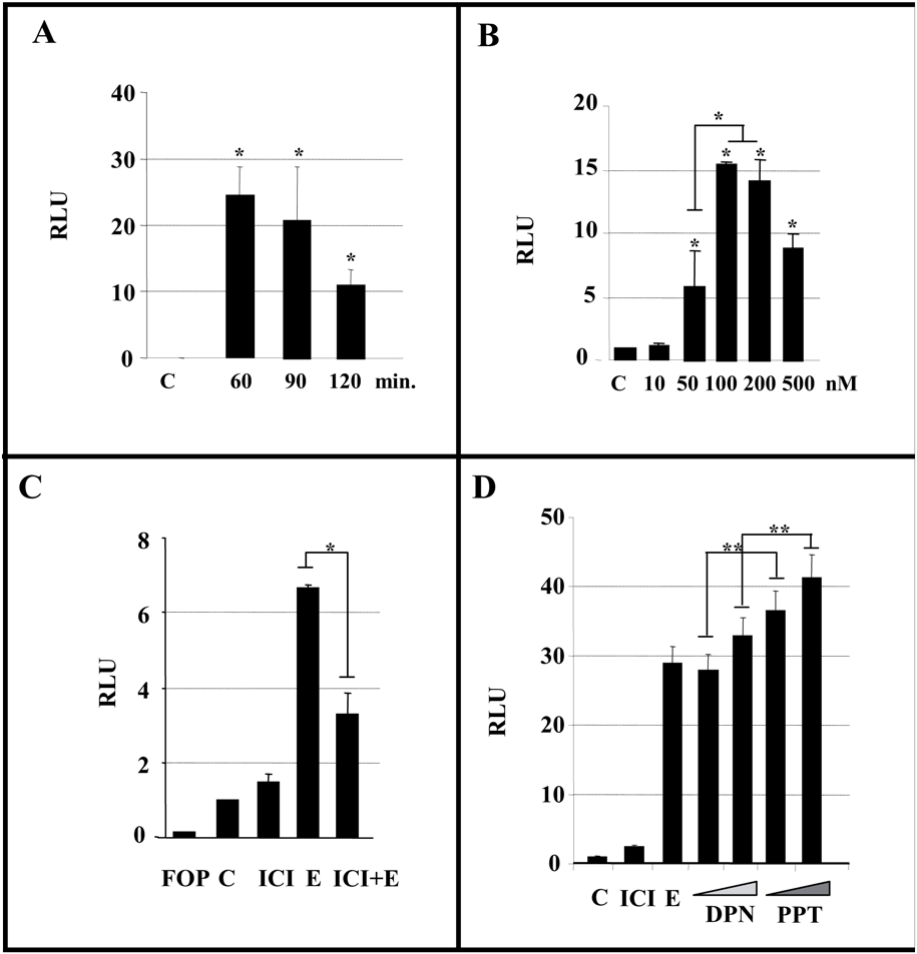
Estradiol augments TCF/LEF-dependent transcription in N2a-m cells. N2a-m cells were transfected with the TOPFlash or FOPFlash reporter plasmid with EGFP-pCDNA3, and the cells were then treated with estradiol and other test compounds for the different times and at the different concentrations indicated. (A–B), Soluble extracts from estradiol treated cells were obtained and *luciferase* activity was measure as indicated in the Methods. The normalized data were expressed in relative light units (RLU) compared to the control solvent, and the response was maximal at 60 min using 100 nM. (C).The effect of estradiol can be prevented by prior exposure to the ER antagonist ICI 182780 10 µM for 2 hr. (D), Specific agonists of each estrogen receptor (PPT: ERα agonist, and DPN: ERβ agonist, at two concentrations, 5 and 10 nM) also induced TCF/LEF-mediated transcription although PPT was more efficient than DPN. The graphs show the normalized *luciferase* activity from at least three independent experiments. The P value from the Student's t-test was * (P≤0.05), ** (P≤0.01). The single * or ** compares data to control whereas the bar between different points shows statistical differences between them.

Even though accumulation of β-catenin in the cell nucleus could not be perceived at the biochemical level, we assessed whether estradiol might induce β-catenin mediated transcriptional activation. For this purpose we transfected N2a-m cells with a TCF-luciferase reporter (TOPFlash) in combination with an EGFP reporter. When estradiol (100 nM) was added after cell transfection, luciferase activity increased 24±4 fold (n = 4, Figure 3D), reaching a maximum 60–90 minutes after hormonal exposure (Figure 3A). The estradiol-induced increase in luciferase activity was time and concentration-dependent (Figure 3A–B). Moreover, the transcriptional activity depended on ERs since it was drastically reduced when the ER antagonist ICI 182780 was applied in conjunction with estradiol (Figure 3C). In parallel, when we transfected N2a-m cells with a mutated form of the TCF reporter (FOPFlash) we were unable to detect *luciferase* activity (Figure 3C).

To determine whether this transcriptional activation was associated with ER α or ER β, we used two specific agonists of either the α or β receptor isoforms. Both 4,4′,4″-(propyl-[(1)H]-pyrazole-1,3,5-triyl) trisphenol (PPT, α-selective) and 2,3-bis (4-hydroxyphenyl) propionitrile (DPN (β-selective) agonists increased transcriptional activity in N2a-m cells, although the activity mediated by PPT was more prominent that that mediated by DPN, suggesting that ER α is more effectively coupled to this novel pathway (Figure 3D). As a positive control of this TCF-reporter, we took advantage of the fact that this neuronal cell responds to some Wnt proteins and thus, β-catenin stabilization was detected when N2a-m cells were exposed to Wnt3a (Supplementary Figure S3). When we exposed cells to Wnt3a after TOPFlash transfection, the transcriptional activation obtained in response to Wnt3a was similar to that obtained after estradiol treatment (Supplementary Figure S3).

### The expression of a TCF-β-galactosidase reporter, as well as an engrailed-1 promoter luciferase reporter, is modulated by estradiol in primary neurons

**Figure 4:**
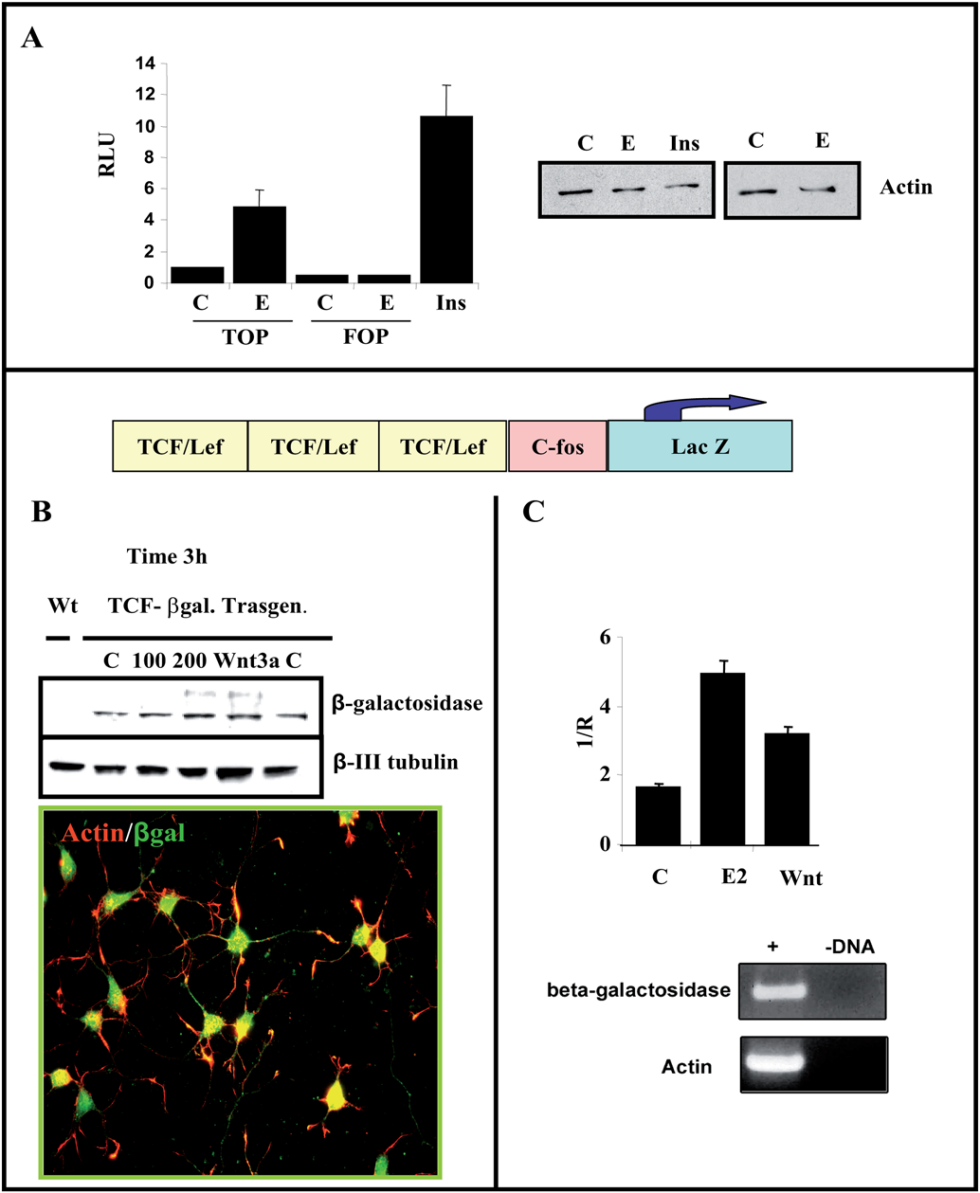
Estradiol increase TCF/LEF-dependent transcription in cortical neurons. (A)-Cortical neurons from E18 embryos were nucleofected with TOPFlash or FOPFlash reporter plasmids and luciferase activity was analyzed after 2DIV. Estradiol treatment (60 min, 100 nM) selectively increased transcription from the TOPFlash reporter plasmid compared to FOPFlash, which shows no activity. Insulin treatment (5 µg/ml) was used as a control of induction. (B–C)- Expression of the LacZ gene in response to estradiol in transgenic mice. The scheme represents the lacZ transgene under the control of three consensus TCF/LEF-binding motifs upstream of the c-fos promoter, as described in *Materials and Methods*. (B)-Upper panel. Total extracts of cortical neurons (2DIV) were obtained from a TCF/LEF-lacZ transgenic mouse (see Materials and Methods) and the β-galactosidase (β-gal) expression was assessed in western blots after estradiol treatment (100–200 nM) for 3 h. Wnt3a (20 ng/ml) was used as a control of TCF-mediated induction. A slight increase in β-gal protein was observed after exposure to estradiol, as with recombinant Wnt3a protein. (B), Lower panel. Basal expression of β-galactosidase in neurons from transgenic mice was assessed by immunocytochemistry using specific antibodies against β galactosidase (green) and Phalloidin-labelled with Alexas 549. (C), Alternatively, after treatment with estradiol or Wnt3a, total Lac Z expression was quantified by RT-PCR using specific β-gal oligonucleotides and using actin (a housekeeping gene) as an internal standard (see *Methods*). The amplification of both genes was analyzed on agarose gels and the graph represents the normalized data obtained from the Lightcycler analysis. Both treatments clearly increase transcriptional activity when compared to controls.

To determine whether estradiol can regulate the expression of a reporter controlled by TCF in primary neurons, we used two complementary approaches. First, we transfected TOPFlash or FOPFlash into primary neurons (Figure 4A) and after TOPFlash transfection, we detected an increase in luciferase activity (4.8±1.2 folds; n = 3) in the presence of estradiol (100 nM), which was not induced after FOPFlash transfection (Figure 4A). High concentrations of insulin (ITS supplements) also produced a 10-fold increase in these cells as a positive control (Figure 4A). In a second approach, we obtained primary cultures of cortical neurons from TCF-βgal transgenic mice, a colony designed to analyze the activation of the Wnt pathway in vivo [21]. We detected β-galactosidase expression in almost all cortical neurons of TCF-βgal transgenic mice (Figure 4B) and thus, we analyzed the amount of β-galactosidase after exposure to either estradiol or Wnt3a. Both estradiol and Wnt3a produced a similar moderate increase in β-galactosidase, assessed in western blots (Figure 4B). This finding was confirmed by quantitative RT-PCR in parallel experiments. Hence, in total RNA obtained from cultured cortical neurons, a similar increase in the expression of the LacZ-transgene was detected by RealTime RT-PCR after exposure to Wnt3a or estradiol when normalized to actin mRNA expression (Figure 4C).

**Figure 5:**
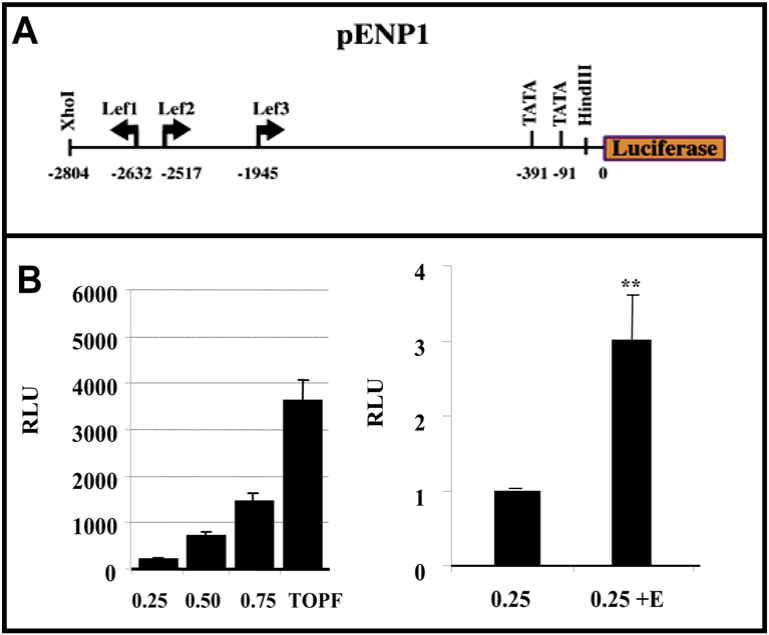
The engrailed 1-*luciferase* construct responds to estradiol. The scheme represents the structure of the 2.8 kB of construct containing the proximal region of endogenous *engrailed* 1 promoter bind to luciferase reporter. N2a-m cells were transfected with the pENP1-luciferase reporter plasmid (*250*, *200 and 750 ng*) (represented as 0.25, 0.5, 0.75), which contains three LEF-1 sites (see upper panel). The Lower panel shows a comparison of the induction of TOPFlash (*750 ng*) and pENP-luc in this cell line. Although basal levels of luciferase activity are lower in the pENP1-luc reporter plasmid, estradiol induces this activity to 3-fold that of the control levels, as shown in the right panel. The graph in B shows the normalized *luciferase* activity from at least three independent experiments. The P value from the Student's t-test was ** (P≤0.01) when compared with control data.

As a complementary alternative we try to analyze whether an endogenous promoter may be regulated by estradiol in a similar fashion as Wnt3a. For that purpose we used a construct containing a 2.8 Kb region of the Engrailed-1 promoter that has three LEF-1 sites situated upstream of a luciferase gene pENP1*Luc* [22] (see scheme in Figure 5A). Again luciferase expression was clearly driven by estradiol, although at identical amounts of cDNA the response of TOPFlash was more potent (Figure 5B).

### Does estradiol use a β-catenin/TCF transcription system similar to that used by Wnt?

**Figure 6:**
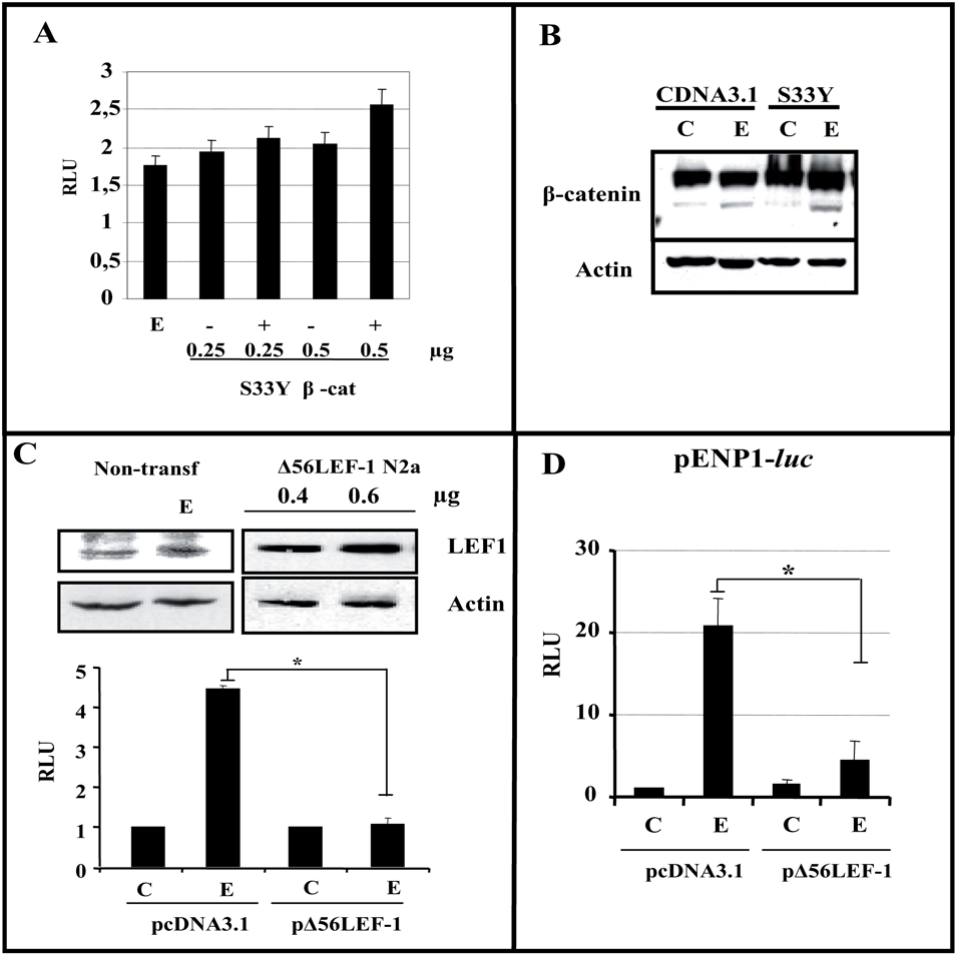
Interaction of β-catenin/LEF-1 mediates the transcriptional capacity of estradiol. (A)-β-catenin/TCF estradiol-mediated transcription depends on the phosphorylation of β-catenin. N2a-m cells were transfected with different amounts of the S33Y-βcatenin (S33Y-βcat) expression plasmid as indicated (*250 or 500 ng*). (B)-β-catenin levels in total extracts from cells transfected with S33Y-βcat or the empty cDNA3.1 plasmid, representative of the analysis of lanes (0.5+); and (0.5−) in A. Under the high levels of S33Y-βcat expression estradiol was virtually unable to further induce reporter expression. No statistical differences are founded between the bars data from different experiments. (C)-Interaction of β-catenin with TCFs is essential for estradiol to induce gene transcription through TCF sites. Endogenous LEF-1 protein levels remain unchanged after estradiol treatment for 60–90 min, as seen with the anti-N terminal LEF-1 antibody. Expression of the Δ56LEF-1 protein was detected in western blots after transfection of increasing amounts of plasmid (*400 or 600 ng*) using an antibody against the HMG box region of LEF-1. The overexpression of a LEF-1 mutant construct (Δ56LEF-1) prevents estradiol from inducing expression from the TOPFlash reporter plasmid when compared with *mock*-transfected cells. (D)-Δ56LEF-1 reduces estradiol induced luciferase expression from pENP1*-luc*. The overexpression of Δ56LEF-1 construct reduced the luciferase activity induced by estradiol from the pENP1-Luc plasmid, when compared with *mock*-transfected cells (*empty*-pcDNA3). Estradiol (E) induced luciferase activity to 8–15 fold that of the control levels (C and E), in the pENP1-luc reporter. However, the expression of Δ56LEF-1 diminished this induction (compare pCDNA3+E *versus* Δ56+E). In both cases (C–D), the graphs show the normalized *luciferase* activity (RLU) from at least three independent experiments. The P value from the Student's t-test was * (P≤0.05).

The mechanism by which Wnt modulates β-catenin-mediated transcription is sensitive to the phosphorylation of β-catenin as well as to β-catenin/TCF binding (reviewed in [17]). Thus, when we transfected N2a-m cells with different amounts of a constitutively active mutant of β-catenin (S33Y), the transcriptional activity in extracts of these cells was almost maximal and it was virtually insensitive to the addition of estradiol (15–20%), when compared with 5–12 folds induction (control or mock-transfected cells *versus* estradiol) (Figure 6A *versus* Figure 3B–C). In all these experiments, the total amount of β-catenin after transfection and treatment was controlled to ensure that it did not influence the results (Figure 6B). Hence, these data strongly suggested that estradiol-mediated transcription requires the formation of a β-catenin/TCF complex. Indeed, when a version of LEF-1 that is truncated in the β-catenin binding region (Δ56LEF-1) [23] was transiently transfected into N2a-m neuroblastoma cells in combination with TOPFlash, estradiol did not drive *luciferase* expression (Figure 6C), whereas N2a-m-*mock* transfected cells responded to estradiol as previously shown.

Similarly, when we transiently transfected into N2a-m neuroblastoma cells with Δ56LEF-1 in combination with pENP1 *Luc*, the *luciferase* expression estradiol-mediated was severely reduced by the expression of LEF1-mutant (Figure 6D).

### Are β-catenin/TCF-DNA complexes activated by estradiol?

**Figure 7:**
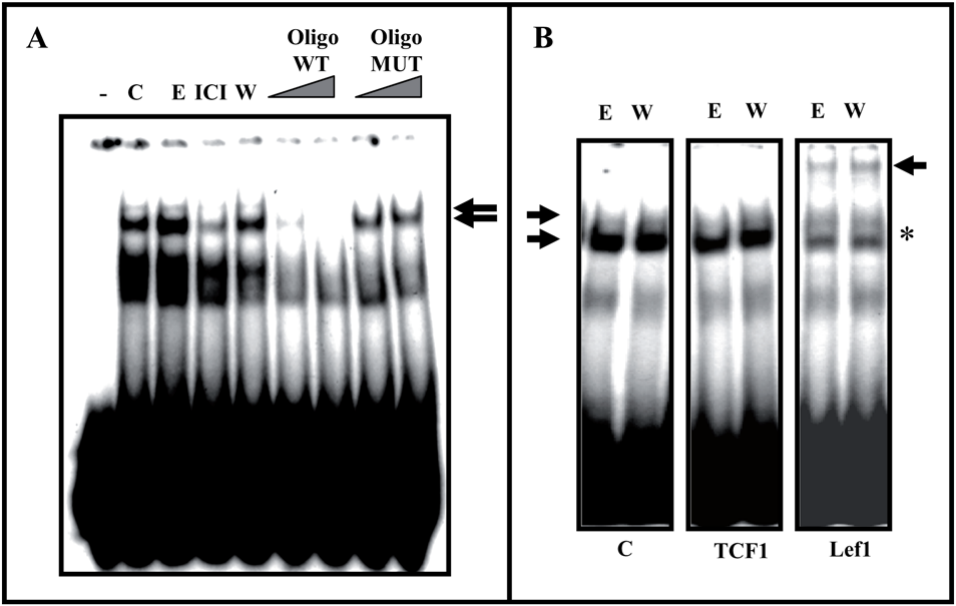
The LEF-1-DNA complex is detected in nuclear protein extracts and it is modulated by estradiol. (A)-Identification of the specific band by competition with non-labeled wt oligonucleotides. Nuclear proteins were obtained from N2a-m cells treated for 30 minutes with estradiol, ICI or Wnt3a, enabling specific TCF-DNA complexes to be detected. In control nuclear extracts the presence of a pre-established TCF-DNA complex was detected, which augmented slightly in the presence of estradiol and that decreased upon ICI treatment. The arrow indicates the complex. (B)-Analysis of the identity of the TCF-DNA complex. Antibodies against TCF1, LEF-1, or irrelevant IgGs (C) were added to the nuclear protein extracts and modification of the DNA/protein complex was evaluated. In addition, antibodies against TCF3 or ERα were used in parallel experiments, without producing any modification of this pattern (Supplementary Figure S4). Only in the case of the anti-LEF-1 antibody was a more slowly migrating band observed. The arrow with an asterisk indicates the appearance of a higher molecular weight complex.

To determine whether the estradiol-mediated transcriptional activation detected in our study relies on similar complexes to those described for Wnt, we assayed the DNA-protein complexes with TCF-binding sequence in nuclear extracts from N2a-m cells (*EMSA assay*). We detected a DNA-protein complex that was more evident after estradiol or Wnt3a treatment (Figure 7). Moreover, the formation of this complex was impaired by pre-incubation with the ER antagonist ICI 182780 and the detection of the complex could be competed with unlabelled TCF-oligonucleotide but not by a mutant TCF oligonucleotide (Figure 7 A).

Four members of TCF family have been described (TCF1, LEF-1, TCF3, and TCF4), the first three of which are found in neuronal tissues [24]. To determine which of these may participate in the TCF/DNA complex described in our system, we pre-incubated the nuclear extracts with specific antibodies against TCF1, LEF-1 or TCF3, as well as with antibodies against ERα or irrelevant IgGs. The antibody against LEF-1 abolished the formation of the DNA-protein complex while the antibodies against TCF1, TCF3 and ERα did not appreciably modify the DNA-protein complex (Figure 7B and supplementary Figure S4).

### Are the genes regulated by estradiol/β-Catenin/LEF-1 similar to those regulated by Wnt/β-Catenin/TCFs?

**Figure 8:**
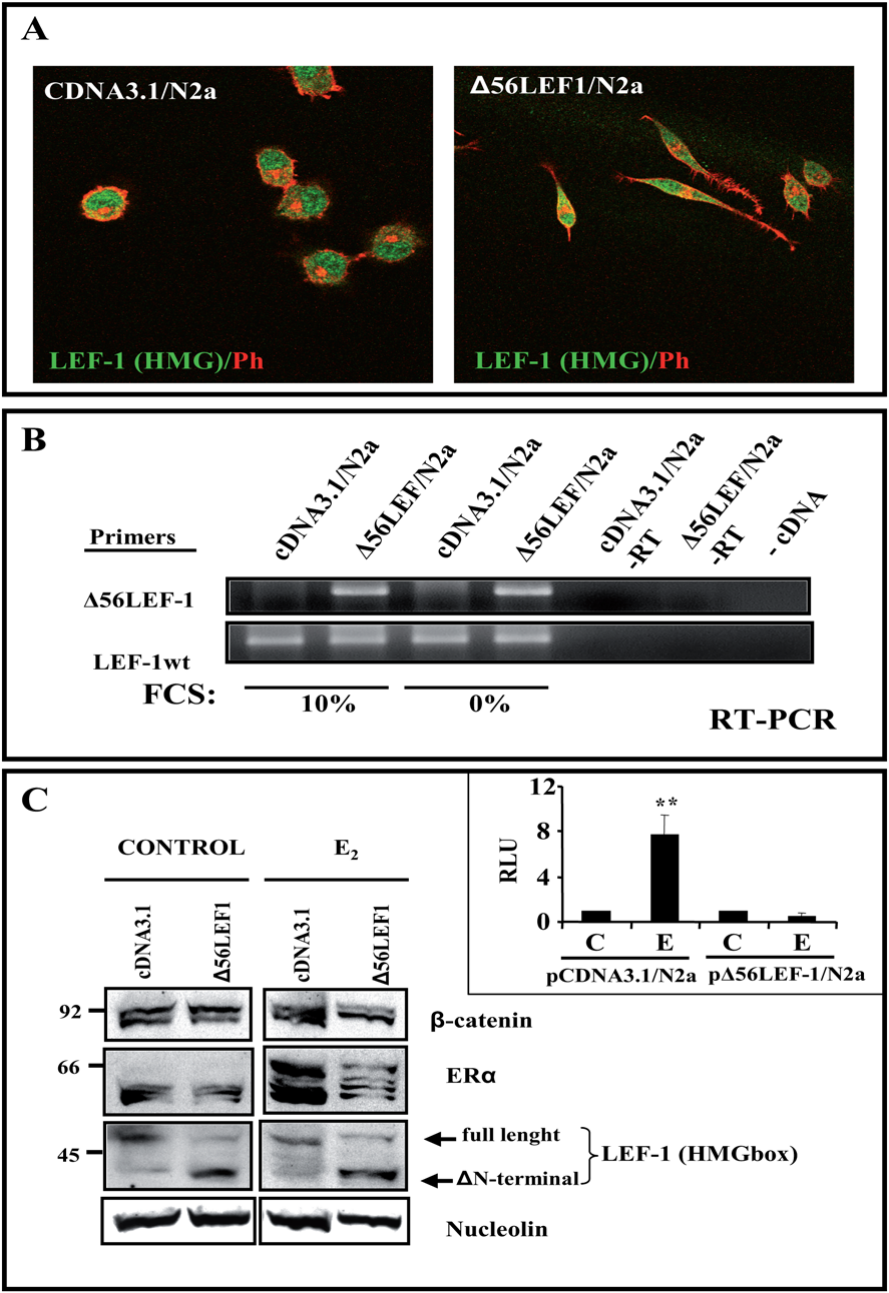
Generation of a stable N2a-m cell line expressing the Δ56LEF-1 protein. N2a-m cells were co-transfected with Δ56LEF-1 or the empty pCDNA3 vector containing the Puromycin resistance gene (for details see *Methods*). (A)- N2a-m-Δ56LEF-1 expression was observed by dual immunocytochemistry using an LEF-HMGbox antibody (green) and Phalloidin (red). Note the morphological changes associated with the expression of LEF-1 mutant. (B)- RNA from the different stable N2a-m cell lines was obtained and the RT-PCR products were analyzed in agarose gels. Expression of the Δ56LEF-1 plasmid was tested using oligos specific to the Δ56LEF-1 plasmid, in parallel with specific oligos recognizing endogenous LEF-1 protein (LEF-1-wt) as controls. Note that no significant differences in plasmid expression were observed between cells growing in 10% FCS compared to those grown in the absence of FCS. (C)-Protein expression was determined to test for the presence of the mutant LEF-1 protein in N2a-m cells. Nuclear extracts were prepared from control and estradiol treated cells, and little accumulation of β-catenin was detected after exposure to estradiol although the estrogen receptor does enter the nucleus. The LEF-HMG box antibody allows us to differentiate full-length LEF-1 from Δ56 LEF-1 in western blots. Nucleolin levels were used as an internal control. The right insert represents the luciferase activity (RLU) of both stable cell lines. A functional analysis was performed to check the loss of estradiol induction over TOPFlash in these cells, as previously reported for the transient transfection (Figure 6C). The graph shows the normalized *luciferase* activity from at least three independent experiments. The P value from the Student's t-test was ** (P≤0.01) when compared with control data.

Together, the data obtained so far suggested that estradiol might activate a β-Catenin/LEF-1 complex that is similar to that activated by Wnt in other systems. Thus, to shed further light on this new molecular mechanisms, we took advantage of the fact that the Δ56LEF-1 construct may act as a dominant-negative construct and we generated N2a-m cells stably transfected with Δ56LEF-1 (Figure 8A). This cell line presented a more differentiated morphology, with bipolar extension of two processes (Figure 8A).

As an internal control, we analyzed the presence of the Δ56LEF-1 as well as the endogenous LEF-1. The expression of Δ56LEF-1 in this cell line was not drastically impaired, and it was maintained at an mRNA and protein ratio of at least 1∶1 when compared with LEF-1, in a cell line *mock*-transfected with the empty pcDNA3.1 vector (Figure 8B and C). The dominant-negative activity of the LEF-1 mutant was observed when the cell line was transiently transfected with TOPFlash. The expression of *luciferase* was still blocked when the cells were incubated with estradiol, as described previously (Figure 8C). Even though, estrogen receptor and β-catenin may be detected in the nuclear fraction of these transfected cells (Figure 8C).

Subsequently, stably transfected N2a-m cells were utilized to obtain the total RNA, either from *empty* pcDNA3/N2a-m (group **A**) or Δ56LEF-1/N2a-m (group **B**). In both cases, cells were treated with estradiol or Wnt3a for 60 minutes and then total RNA was purified. Gene expression from the two stable cell types (*A and B*) was then analyzed using the Applied Biosystems Mouse Genome Survey Microarray (*Applied Biosystems*).

**Figure 9:**
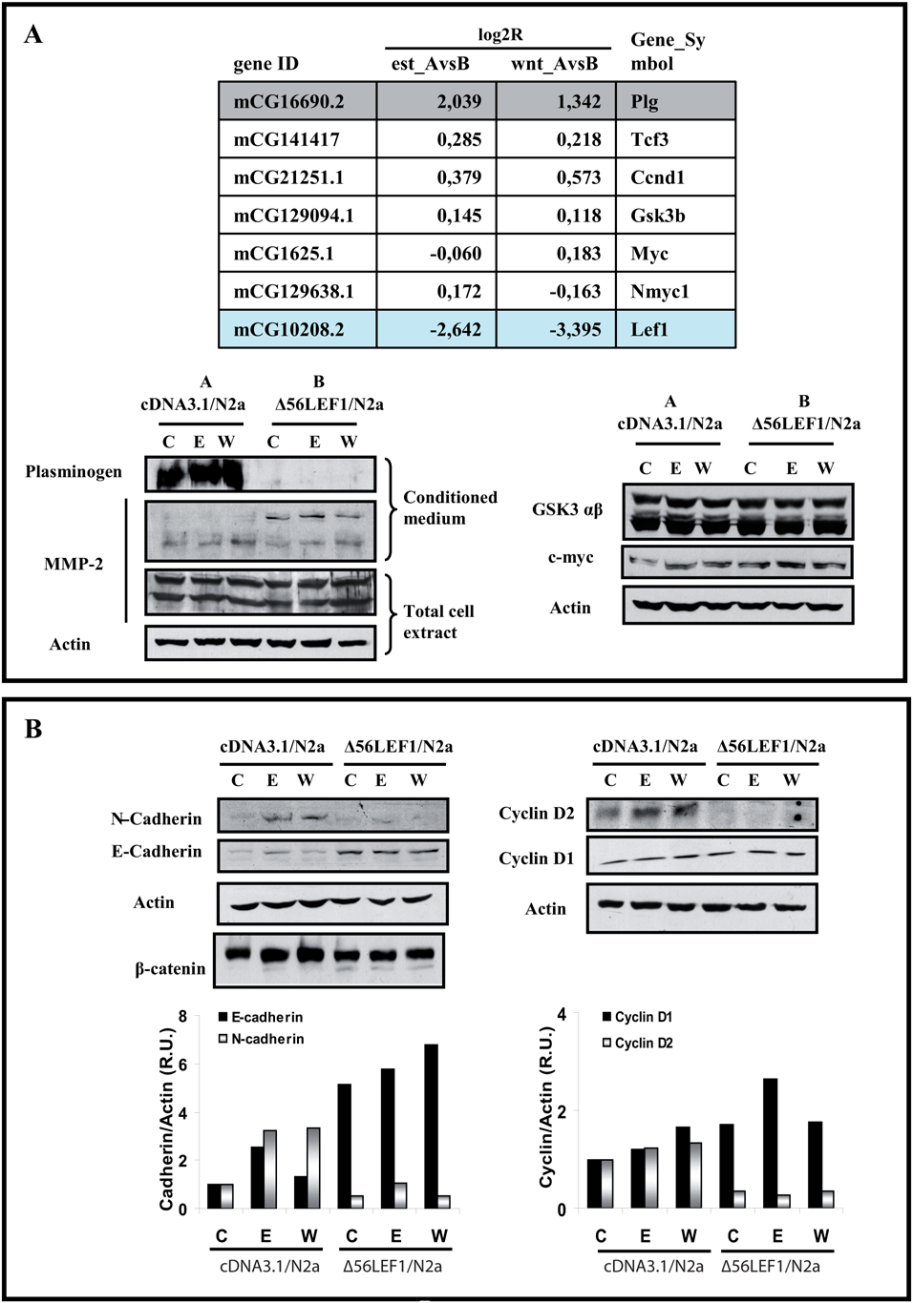
Gene expression of cDNA/N2a-m and Δ56LEF-1/N2a-m cells after exposure to estradiol. (A)- Gene expression profile in cDNA/N2a-m and Δ56LEF-1/N2a-m stables cell lines. The upper panel reflects the gene induction of some selected genes, in microarray analysis of RNA collected after a 45 min exposure to estradiol or Wnt3a to detect the early response. Data is expressed as log_2_R from cDNA/N2a-m cells, that we denoted as group A, and Δ56LEF-1/N2a-m cells, that we denoted as group B. The effect of the treatment was compared between the two stable cell lines (A vs B) (see “Table 1” for a more complete list of the annotated genes). As seen, in the panel we selected some “putative Wnt-regulated genes”, such as *Tcf3*, *Ccnd1* (cyclin D1), *GSK3b*, *Myc* and *LEF-1*, to give some examples of the results in our arrays. We detected changes at the protein level only in *Plg*, although there were several genes whose expression varied. For example, the levels of plasminogen RNA were much higher in group B than group A (ratio AvsB≥1), and the expression of LEF-1 was higher in Δ56LEF-1 due to the mutant expression (ratio AvsB≤−1). The western blots below are verifications of these differences at the protein level. Among other proteins that did not change between the groups of cells were GSK3 β or myc (see western blots on the right). MMP-2 was tested although it did not display a change in its RNA levels and there was no difference in the total cell extracts. Interestingly, when conditioned medium was prepared, more pro-active MMP-2 (and less active protein) was seen in Δ56LEF-1 cells. [Gene_Symbol: *Plg* (plasminogen), *Tcf3*, *Ccnd1* (cyclin D1), *GSK3b*, *Myc* and *LEF1*]. (B)- Estradiol induction of N-cadherin and cyclin D2 may be affected by expression of the Δ56LEF-1 protein. Total cell extracts from cDNA/N2a-m cells (group A) and Δ56LEF-1/N2a-m cells (group B) were collected 24 h after estradiol or Wnt3a treatment to analyze several known Wnt or estrogen target genes. As seen in western blots, estradiol upregulated E-cadherin, N-cadherin and cyclin D2 expression in group A cells. N-cadherin and cyclin D2 were also upregulated by Wnt in group A cells. In contrast, E-cadherin expression was not Wnt responsive. The regulatory effects of estradiol on E-cadherin, N-cadherin and cyclin D2 expression were lost when Δ56LEF-1 is expressed, as seen in group B cells. In the case of E-cadherin, the loss of functional LEF-1, which acts as a known gene repressor, implies higher protein levels even without stimulation. In contrast, levels of actin or cyclin D1 remained unchanged.

Genes were normalized with respect to control untreated cells, and divided in different groups. From these groups, we only consider those with differences when compared group **A** versus group **B**, for each treatment. From these we selected those with a log_2_R higher or lower than **1**, being 102 genes upregulated and 121 genes downregulated by estradiol; and 137 genes upregulated and 166 genes downregulated by Wnt3a. **Table 1** described the list of the annotated genes present in the protein bank with the *Ref Seq* and the *Gene Name*. In contrast, the genes modified only after estradiol treatment or only after Wnt3a treatment that presented no significant differences between **A** and **B** were discarded. Initially, for the confirmation of the putative deferential response of both cells (A vs B) we selected two genes presented in both arrays that were modified by Wnt as well as by estradiol (Plasminogen and LEF-1). A final validation was performed using specific antibodies against the selected proteins (Figure 9 and 8C). Thus, plasminogen expression in Δ56LEF-1 cells was 2.5 fold higher, although these initial differences were not considerably augmented by treatment with Wnt or estradiol. Similarly, the endogenous LEF-1 was reduced by the presence of the mutant version of this protein (Figure 8). In addition we selected some genes, previously reported by other laboratories as Wnt targets , such as TCF's, Cyclin D, Myc, MMP's (for updated, please check, The Wnt Homepage, *at* http://www.stanford.edu/~rnusse/pathways/targets.html).

**Table 1:** Results of the Microarray analysis of gene transcription.

UPREGULATED:		
RefSeq_NM	est_A vs B	Gene_Name
NM_007990.1	2,739	Finkel-Biskis-Reilly murine sarcoma virus (FBR-MuSV) ubiquitously expressed (fox derived)
NM_133198.1	2,676	liver glycogen phosphorylase
NM_130888.1	2,457	nuclear RNA export factor 7
NM_025703.2	2,439	RIKEN cDNA 3930402F23 gene
NM_183271.1	2,181	RIKEN cDNA 1700049M11 gene
NM_008877.2	2,039	**plasminogen**
NM_007407.2	2,034	adenylate cyclase activating polypeptide 1 receptor 1
D87975.1	1,994	serine (or cysteine) proteinase inhibitor, clade F, member 1
NM_019703.2	1,890	phosphofructokinase, platelet
NM_010480.4	1,847	heat shock protein 1, alpha
NM_054095.1	1,846	EF hand calcium binding protein 2
NM_013779.1	1,701	melanoma antigen, family L, 2
NM_021389.3	1,653	SH3-domain kinase binding protein 1
NM_009529.1	1,509	Xlr-related, meiosis regulated
NM_133362.1	1,491	erythroid differentiation regulator 1
NM_183294.1	1,487	cyclin-dependent kinase-like 1 (CDC2-related kinase)
NM_007833.1	1,462	decorin
BC053970.1	1,337	NADH dehydrogenase (ubiquinone) 1, alpha/beta subcomplex, 1
NM_008144.3	1,335	Bernardinelli-Seip congenital lipodystrophy 2 homolog (human)
NM_033325.1	1,332	lysyl oxidase-like 2
NM_175027.2	1,321	cDNA sequence BC022692
NM_146034.1	1,316	meningioma expressed antigen 6 (coiled-coil proline-rich)
NM_177752.2	1,313	hypothetical protein 6820428D13
NM_025437.2	1,298	eukaryotic translation initiation factor 1A, Y-linked
NM_019768.2	1,291	mortality factor 4 like 2
NM_008590.1	1,251	mesoderm specific transcript
NM_019647.3	1,247	ribosomal protein L21
NM_031174.2	1,246	Down syndrome cell adhesion molecule
NM_009093.1	1,237	ribosomal protein S29
NM_008200.1	1,225	histocompatibility 2, D region locus 4
NM_008033.2	1,222	farnesyltransferase, CAAX box, alpha
NM_026712.1	1,216	RIKEN cDNA 0610030H11 gene
NM_183203.1	1,202	hypothetical protein 9130430E04
NM_145628.2	1,200	ubiquitin specific protease 11
NM_207204.1	1,188	RIKEN cDNA 4930519N13 gene
NM_025695.2	1,178	SMC6 structural maintenance of chromosomes 6-like 1 (yeast)
NM_008183.2	1,177	glutathione S-transferase, mu 2
NM_145526.1	1,173	purinergic receptor P2X, ligand-gated ion channel, 3
NM_012035.1	1,136	transient receptor potential cation channel, subfamily C, member 7
NM_010206.1	1,135	fibroblast growth factor receptor 1
NM_146035.1	1,122	mannoside acetylglucosaminyltransferase 2
NM_175930.2	1,118	Rap guanine nucleotide exchange factor (GEF) 5
NM_013819.1	1,111	histocompatibility 2, M region locus 3
NM_008666.1	1,094	myelin transcription factor 1-like
NM_029823.1	1,092	RIKEN cDNA 6530401D17 gene
NM_172758.1	1,090	cDNA sequence BC031853
AK040457.1	1,085	interleukin 27 receptor, alpha
NM_194355.1	1,079	spire homolog 1 (Drosophila)
NM_007881.4	1,072	dentatorubral pallidoluysian atrophy
NM_025522.1	1,064	dehydrogenase/reductase (SDR family) member 7
NM_008549.1	1,063	mannosidase 2, alpha 1
NM_010545.2	1,058	Ia-associated invariant chain
NM_008810.2	1,058	pyruvate dehydrogenase E1 alpha 1
NM_175007.1	1,044	amphiphysin
NM_028270.2	1,029	aldehyde dehydrogenase 1 family, member B1
NM_008737.1	1,024	neuropilin
NM_008666.1	1,023	myelin transcription factor 1-like
NM_025932.1	1,016	synapse associated protein 1
NM_010195.1	1,006	G protein-coupled receptor 49

DOWNREGULATED:		
RefSeq_NM	est_AvsB	Gene_Name
NM_019932.1	−3,893	chemokine (C-X-C motif) ligand 4
NM_010703.2	−2,642	**lymphoid enhancer binding factor 1**
NM_013492.1	−2,293	clusterin
NM_011702.1	−2,053	vasoactive intestinal polypeptide
NM_010685.2	−1,992	lysosomal membrane glycoprotein 2
NM_011333.1	−1,986	chemokine (C-C motif) ligand 2
NM_010658.2	−1,809	v-maf musculoaponeurotic fibrosarcoma oncogene family, protein B (avian)
NM_010515.1	−1,705	insulin-like growth factor 2 receptor
AK033214.1	−1,677	G elongation factor
BC053489.1	−1,557	insulin-like growth factor 2
NM_023422.1	−1,438	histone 1, H2bc
AK027922.1	−1,427	glutathione S-transferase omega 1
NM_177708.3	−1,420	reticulon 4 receptor-like 1
NM_175395.2	−1,418	RIKEN cDNA 1700001A24 gene;RIKEN cDNA 6330578E17 gene
NM_053273.1	−1,410	tweety homolog 2 (Drosophila)
NM_009344.1	−1,395	pleckstrin homology-like domain, family A, member 1
NM_011844.3	−1,349	monoglyceride lipase
NM_009776.1	−1,346	serine (or cysteine) proteinase inhibitor, clade G, member 1
NM_021274.1	−1,339	chemokine (C-X-C motif) ligand 10
NM_021897.1	−1,337	transformation related protein 53 inducible nuclear protein 1
NM_138683.1	−1,303	thrombospondin type 1 domain containing gene
NM_010789.1	−1,292	myeloid ecotropic viral integration site 1
NM_016867.1	−1,291	semaF cytoplasmic domain associated protein 2
BC058269.1	−1,290	RAR-related orphan receptor beta
NM_007635.2	−1,279	cyclin G2
NM_015818.1	−1,275	heparan sulfate 6-O-sulfotransferase 1
NM_026416.2	−1,261	S100 calcium binding protein A16
NM_145825.1	−1,260	centrin 4
AK035160.1	−1,252	heart and neural crest derivatives expressed transcript 2
NM_028535.1	−1,251	RIKEN cDNA 1700049E17 gene
AK031672.1	−1,246	DNA segment, Chr 11, ERATO Doi 498, expressed
NM_178405.2	−1,233	ATPase, Na+/K+ transporting, alpha 2 polypeptide
NM_172854.1	−1,213	RIKEN cDNA 4932431K08 gene
NM_009242.1	−1,180	secreted acidic cysteine rich glycoprotein
NM_021537.2	−1,172	serine/threonine kinase 25 (yeast)
NM_008250.1	−1,167	H2.0-like homeo box gene
NM_008538.2	−1,167	myristoylated alanine rich protein kinase C substrate
NM_026793.1	−1,166	myc target 1
NM_011353.1	−1,163	small EDRK-rich factor 1
NM_026358.1	−1,160	RIKEN cDNA 4930583H14 gene
NM_009726.2	−1,158	ATPase, Cu++ transporting, alpha polypeptide
NM_194268.2	−1,155	one cut domain, family member 2
NM_011295.2	−1,142	ribosomal protein S12
NM_175103.2	−1,135	RIKEN cDNA 1110025L05 gene
NM_025836.1	−1,130	mannose-6-phosphate receptor binding protein 1
NM_025692.1	−1,127	ubiquitin-activating enzyme E1-domain containing 1
NM_025394.1	−1,126	translocase of outer mitochondrial membrane 7 homolog (yeast)
NM_028279.1	−1,125	N-acetylated alpha-linked acidic dipeptidase 2
NM_133780.2	−1,125	translocated promoter region
NM_013660.1	−1,120	sema domain, immunoglobulin domain (Ig), transmembrane domain (TM) and short cytoplasmic domain, (semaphorin) 4D
NM_013692.1	−1,120	TGFB inducible early growth response 1
NM_010357.1	−1,119	glutathione S-transferase, alpha 4
NM_018865.1	−1,108	WNT1 inducible signaling pathway protein 1
NM_010317.2	−1,107	guanine nucleotide binding protein (G protein), gamma 4 subunit
NM_173788.2	−1,101	natriuretic peptide receptor 2
NM_175238.4	−1,089	DNA segment, Chr 2, ERATO Doi 145, expressed
NM_011313.1	−1,088	S100 calcium binding protein A6 (calcyclin)
NM_025735.1	−1,080	microtubule-associated protein 1 light chain 3 alpha
NM_178751.2	−1,080	RIKEN cDNA A730041O15 gene
NM_030037.1	−1,078	motile sperm domain containing 3
NM_017367.2	−1,078	cyclin I
NM_133218.1	−1,077	glucocorticoid induced gene 1
AK010069.1	−1,075	DNA segment, Chr 7, Wayne State University 87, expressed
NM_026033.1	−1,074	ocular development associated gene
NM_021398.1	−1,070	solute carrier family 43, member 3
NM_174988.2	−1,063	cadherin 22
NM_026111.2	−1,043	RIKEN cDNA 1810019P04 gene
NM_012057.1	−1,042	interferon regulatory factor 5
AK076923.1	−1,042	protein phosphatase 1B, magnesium dependent, beta isoform
NM_133859.1	−1,040	olfactomedin-like 3
NM_026565.2	−1,038	RIKEN cDNA 9430083G14 gene
NM_133942.1	−1,037	expressed sequence AA960558
AK003039.1	−1,030	glycolipid transfer protein
NM_008967.1	−1,022	prostaglandin I receptor (IP)
NM_023649.1	−1,020	Usher syndrome 1C homolog (human)
NM_025946.2	−1,013	RIKEN cDNA 2010100O12 gene
NM_009113.2	−1,010	S100 calcium binding protein A13
NM_027285.1	−1,009	RIKEN cDNA 1700029I01 gene
NM_172296.1	−1,009	doublesex and mab-3 related transcription factor like family A2
NM_009814.1	−1,007	calsequestrin 2
NM_013765.1	−1,005	ribosomal protein S26
NM_011952.1	−1,003	mitogen activated protein kinase 3
NM_010831.1	−1,001	SNF1-like kinase

UPREGULATED:			
RefSeq_NM	Wnt_AvsB	Gene_Symbol	Gene_Name
NM_007990.1	3,603	Fau	Finkel-Biskis-Reilly murine sarcoma virus (FBR-MuSV) ubiquitously expressed (fox derived)
NM_133198.1	2,620	Pygl	liver glycogen phosphorylase
NM_019703.2	2,343	Pfkp	phosphofructokinase, platelet
NM_007407.2	2,294	Adcyap1r1	adenylate cyclase activating polypeptide 1 receptor 1
NM_025703.2	2,270	3930402F23Rik	RIKEN cDNA 3930402F23 gene
NM_183271.1	2,215	1700049M11Rik	RIKEN cDNA 1700049M11 gene
BC053970.1	2,198	Ndufab1	NADH dehydrogenase (ubiquinone) 1, alpha/beta subcomplex, 1
NM_130888.1	2,106	Nxf7	nuclear RNA export factor 7
D87975.1	2,052	Serpinf1	serine (or cysteine) proteinase inhibitor, clade F, member 1
NM_021389.3	1,966	Sh3kbp1	SH3-domain kinase binding protein 1
NM_054095.1	1,910	Efcbp2	EF hand calcium binding protein 2
NM_133362.1	1,797	Erdr1	erythroid differentiation regulator 1
NM_177752.2	1,682	6820428D13	hypothetical protein 6820428D13
NM_172960.1	1,608	Adck5	aarF domain containing kinase 5
AK040457.1	1,501	Il27ra	interleukin 27 receptor, alpha
NM_008595.1	1,446	Mfng	manic fringe homolog (Drosophila)
NM_033325.1	1,445	Loxl2	lysyl oxidase-like 2
NM_145526.1	1,425	P2rx3	purinergic receptor P2X, ligand-gated ion channel, 3
NM_008183.2	1,419	Gstm2	glutathione S-transferase, mu 2
NM_025695.2	1,414	Smc6l1	SMC6 structural maintenance of chromosomes 6-like 1 (yeast)
NM_009369.1	1,414	Tgfbi	transforming growth factor, beta induced
NM_025522.1	1,410	Dhrs7	dehydrogenase/reductase (SDR family) member 7
NM_007997.1	1,399	Fdxr	ferredoxin reductase
NM_207204.1	1,382	4930519N13Rik	RIKEN cDNA 4930519N13 gene
NM_007833.1	1,360	Dcn	decorin
NM_008877.2	1,342	Plg	**plasminogen**
NM_019966.1	1,331	Mlycd	malonyl-CoA decarboxylase
NM_175027.2	1,316	BC022692	cDNA sequence BC022692
NM_080553.1	1,309	Itpr3	inositol 1,4,5-triphosphate receptor 3
NM_010480.4	1,308	Hspca	heat shock protein 1, alpha
NM_029823.1	1,273	6530401D17Rik	RIKEN cDNA 6530401D17 gene
NM_008144.3	1,273	Bscl2	Bernardinelli-Seip congenital lipodystrophy 2 homolog (human)
NM_198031.1	1,266	Tubgcp3	tubulin, gamma complex associated protein 3
NM_008810.2	1,249	Pdha1	pyruvate dehydrogenase E1 alpha 1
NM_008666.1	1,246	Myt1l	myelin transcription factor 1-like
NM_173427.1	1,240	B230308G19Rik	RIKEN cDNA B230308G19 gene
NM_178794.2	1,234	U2af1-rs2	U2 small nuclear ribonucleoprotein auxiliary factor (U2AF) 1, related sequence 2
NM_146034.1	1,234	Mgea6	meningioma expressed antigen 6 (coiled-coil proline-rich)
NM_019552.1	1,229	Abcb10	ATP-binding cassette, sub-family B (MDR/TAP), member 10
NM_178915.2	1,221	AI839735	expressed sequence AI839735
NM_008243.2	1,215	Mst1	macrophage stimulating 1 (hepatocyte growth factor-like)
NM_145482.1	1,175	ORF21	open reading frame 21
NM_025936.1	1,173	Rars	arginyl-tRNA synthetase
NM_028262.1	1,168	D12Ertd771e	DNA segment, Chr 12, ERATO Doi 771, expressed
NM_031174.2	1,164	Dscam	Down syndrome cell adhesion molecule
NM_023304.1	1,149	Fgf22	fibroblast growth factor 22
NM_025932.1	1,139	Syap1	synapse associated protein 1
NM_013778.1	1,130	Akr1c13;Akr1c12	aldo-keto reductase family 1, member C13;aldo-keto reductase family 1, member C12
NM_013819.1	1,129	H2-M3	histocompatibility 2, M region locus 3
NM_007407.2	1,126	Adcyap1r1	adenylate cyclase activating polypeptide 1 receptor 1
NM_021273.2	1,122	Ckb	creatine kinase, brain
NM_008033.2	1,119	Fnta	farnesyltransferase, CAAX box, alpha
NM_009529.1	1,115	Xmr	Xlr-related, meiosis regulated
NM_207269.1	1,111	D330050I23Rik	RIKEN cDNA D330050I23 gene
NM_010752.1	1,108	Mad1l1	mitotic arrest deficient 1-like 1
NM_028089.1	1,091	Cyp2c55	cytochrome P450, family 2, subfamily c, polypeptide 55
NM_133224.1	1,090	Atp13a	ATPase type 13A
NM_025656.1	1,089	Sip1	survivor of motor neuron protein interacting protein 1
NM_028002.1	1,088	2310069P03Rik	RIKEN cDNA 2310069P03 gene
NM_010724.1	1,086	Psmb8	proteosome (prosome, macropain) subunit, beta type 8
NM_028131.1	1,081	2610510J17Rik	RIKEN cDNA 2610510J17 gene
NM_009239.1	1,080	Sp4	trans-acting transcription factor 4
NM_025437.2	1,077	Eif1ay	eukaryotic translation initiation factor 1A, Y-linked
NM_183294.1	1,072	Cdkl1	cyclin-dependent kinase-like 1 (CDC2-related kinase)
NM_013779.1	1,070	Magel2	melanoma antigen, family L, 2
NM_021556.2	1,063	Mrps30	mitochondrial ribosomal protein S30
NM_207210.1	1,059	Dyrk4	dual-specificity tyrosine-(Y)-phosphorylation regulated kinase 4
NM_146035.1	1,057	Mgat2	mannoside acetylglucosaminyltransferase 2
NM_144844.1	1,049	Pcca	propionyl-Coenzyme A carboxylase, alpha polypeptide
NM_178389.2	1,045	Gale	galactose-4-epimerase, UDP
NM_012035.1	1,041	Trpc7	transient receptor potential cation channel, subfamily C, member 7
NM_176953.2	1,034	Lig4	ligase IV, DNA, ATP-dependent
NM_008183.2	1,033	Gstm2	glutathione S-transferase, mu 2
NM_178242.2	1,032	Zfp469	zinc finger protein 469
NM_009933.1	1,030	Col6a1	procollagen, type VI, alpha 1
NM_010068.1	1,030	Dnmt3b	DNA methyltransferase 3B
NM_028270.2	1,027	Aldh1b1	aldehyde dehydrogenase 1 family, member B1
NM_172783.1	1,022	Phka2	phosphorylase kinase alpha 2
NM_008590.1	1,020	Mest	mesoderm specific transcript
NM_013761.2	1,017	Srr	serine racemase
NM_029541.1	1,017	6530401D17Rik	RIKEN cDNA 6530401D17 gene
NM_025486.1	1,012	1700006C06Rik	RIKEN cDNA 1700006C06 gene
NM_130864.2	1,012	Acaa1	acetyl-Coenzyme A acyltransferase 1

DOWNREGULATED:			
RefSeq_NM	Wnt_AvsB	Gene_Symbol	Gene_Name
NM_019932.1	−3,616	Cxcl4	chemokine (C-X-C motif) ligand 4
NM_010703.2	−3,395	Lef1	**lymphoid enhancer binding factor 1**
NM_011702.1	−2,102	Vip	vasoactive intestinal polypeptide
NM_010685.2	−2,085	Lamp2	lysosomal membrane glycoprotein 2
NM_011333.1	−1,843	Ccl2	chemokine (C-C motif) ligand 2
AK027922.1	−1,770	Gsto1	glutathione S-transferase omega 1
NM_010515.1	−1,748	Igf2r	insulin-like growth factor 2 receptor
NM_007635.2	−1,679	Ccng2	cyclin G2
NM_013492.1	−1,634	Clu	clusterin
NM_027285.1	−1,585	1700029I01Rik	RIKEN cDNA 1700029I01 gene
NM_177708.3	−1,583	Rtn4rl1	
NM_016867.1	−1,571	Semcap2	semaF cytoplasmic domain associated protein 2
NM_009150.2	−1,552	Selenbp2;Selenbp1	selenium binding protein 2;selenium binding protein 1
NM_177820.2	−1,484	9130218O11Rik	RIKEN cDNA 9130218O11 gene
NM_009726.2	−1,423	Atp7a	ATPase, Cu++ transporting, alpha polypeptide
NM_026416.2	−1,393	S100a16	S100 calcium binding protein A16
NM_023422.1	−1,390	Hist1h2bc	histone 1, H2bc
NM_009760.2	−1,378	Bnip3	BCL2/adenovirus E1B 19kDa-interacting protein 1, NIP3
NM_030037.1	−1,334	Mospd3	motile sperm domain containing 3
NM_010149.2	−1,323	Epor	erythropoietin receptor
NM_172296.1	−1,305	Dmrta2	doublesex and mab-3 related transcription factor like family A2
AK076923.1	−1,305	Ppm1b	protein phosphatase 1B, magnesium dependent, beta isoform
NM_026030.1	−1,298	Eif2s2	eukaryotic translation initiation factor 2, subunit 2 (beta)
NM_194268.2	−1,294	Onecut2	one cut domain, family member 2
NM_008926.2	−1,293	Prkg2	protein kinase, cGMP-dependent, type II
NM_026565.2	−1,291	9430083G14Rik	RIKEN cDNA 9430083G14 gene
NM_025949.1	−1,279	Rps6ka6	ribosomal protein S6 kinase polypeptide 6
NM_009242.1	−1,264	Sparc	secreted acidic cysteine rich glycoprotein
NM_178405.2	−1,256	Atp1a2	ATPase, Na+/K+ transporting, alpha 2 polypeptide
NM_007874.2	−1,237	Dp1	deleted in polyposis 1
NM_026793.1	−1,236	Myct1	myc target 1
NM_177603.1	−1,228	Frat2	frequently rearranged in advanced T-cell lymphomas 2
NM_025836.1	−1,224	M6prbp1	mannose-6-phosphate receptor binding protein 1
NM_028535.1	−1,224	1700049E17Rik	RIKEN cDNA 1700049E17 gene
NM_026358.1	−1,221	4930583H14Rik	RIKEN cDNA 4930583H14 gene
NM_172132.1	−1,218	Jmjd2b	jumonji domain containing 2B
AK005820.1	−1,210	Rabl5	RAB, member of RAS oncogene family-like 5
AK049314.1	−1,193	Trim27	tripartite motif protein 27
NM_017367.2	−1,188	Ccni	cyclin I
NM_009814.1	−1,180	Casq2	calsequestrin 2
NM_009101.1	−1,174	Rras	Harvey rat sarcoma oncogene, subgroup R
AK003039.1	−1,174	Gltp	glycolipid transfer protein
NM_008467.2	−1,172	Kpna4	karyopherin (importin) alpha 4
BC038145.1	−1,172	BC002199	cDNA sequence BC002199
NM_133780.2	−1,168	Tpr	translocated promoter region
NM_177798.3	−1,157	Frs2	fibroblast growth factor receptor substrate 2
NM_008906.2	−1,153	Ppgb	protective protein for beta-galactosidase
NM_015818.1	−1,152	Hs6st1	heparan sulfate 6-O-sulfotransferase 1
NM_009113.2	−1,152	S100a13	S100 calcium binding protein A13
NM_009344.1	−1,148	Phlda1	pleckstrin homology-like domain, family A, member 1
NM_019675.2	−1,148	Stmn4	stathmin-like 4
NM_146141.1	−1,147	1110013G13Rik	RIKEN cDNA 1110013G13 gene
NM_009140.1	−1,146	Cxcl2	chemokine (C-X-C motif) ligand 2
NM_173788.2	−1,145	Npr2	natriuretic peptide receptor 2
NM_021274.1	−1,144	Cxcl10	chemokine (C-X-C motif) ligand 10
AK052456.1	−1,142	D430026P16Rik	RIKEN cDNA D430026P16 gene
NM_153399.1	−1,140	Syne1	synaptic nuclear envelope 1
NM_011756.3	−1,136	Zfp36	zinc finger protein 36
AK083556.1	−1,136	Fst	follistatin
NM_026033.1	−1,122	Odag	ocular development associated gene
NM_031997.2	−1,121	Tmem2	transmembrane protein 2
NM_178772.2	−1,117	B230106I24Rik	RIKEN cDNA B230106I24 gene
NM_011353.1	−1,112	Serf1	small EDRK-rich factor 1
NM_021398.1	−1,110	Slc43a3	solute carrier family 43, member 3
NM_027482.1	−1,095	5730508B09Rik	RIKEN cDNA 5730508B09 gene
NM_133777.1	−1,087	Ube2s	ubiquitin-conjugating enzyme E2S
NM_012057.1	−1,083	Irf5	interferon regulatory factor 5
NM_013660.1	−1,080	Sema4d	sema domain, immunoglobulin domain (Ig), (semaphorin) 4D
NM_146260.1	−1,080	Tmie	transmembrane inner ear
NM_153068.1	−1,075	Ehd2	EH-domain containing 2
NM_138584.1	−1,074	Spg21	spastic paraplegia 21 homolog (human)
NM_011295.2	−1,073	Rps12	ribosomal protein S12
NM_030696.2	−1,071	Slc16a3	solute carrier family 16 (monocarboxylic acid transporters), member 3
NM_130903.2	−1,068	Cd209c	CD209c antigen
NM_020586.1	−1,068	5031400M07Rik	RIKEN cDNA 5031400M07 gene
NM_031997.2	−1,067	Tmem2	transmembrane protein 2
NM_010789.1	−1,065	Meis1	myeloid ecotropic viral integration site 1
NM_011498.2	−1,065	Bhlhb2	basic helix-loop-helix domain containing, class B2
NM_025290.2	−1,063	Tsga2	testis specific gene A2
NM_139144.2	−1,063	Ogt	O-linked N-acetylglucosamine (GlcNAc) transferase (UDP-N-acetylglucosamine:polypeptide-N-acetylglucosaminyl transferase)
NM_009895.2	−1,059	Cish	cytokine inducible SH2-containing protein
NM_011171.1	−1,057	Procr	protein C receptor, endothelial
NM_019880.2	−1,056	Mtch1	mitochondrial carrier homolog 1 (C. elegans)
NM_010658.2	−1,056	Mafb	v-maf musculoaponeurotic fibrosarcoma oncogene family, protein B (avian)
NM_011046.1	−1,054	Furin	furin (paired basic amino acid cleaving enzyme)
NM_011252.2	−1,053	Rbmx	RNA binding motif protein, X chromosome
NM_007585.2	−1,047	Anxa2	annexin A2
NM_025348.1	−1,045	Ndufa3	NADH dehydrogenase (ubiquinone) 1 alpha subcomplex, 3
BC028655.1	−1,044	Solt	SoxLZ/Sox6 leucine zipper binding protein in testis
NM_028279.1	−1,043	Naalad2	N-acetylated alpha-linked acidic dipeptidase 2
NM_018865.1	−1,040	Wisp1	WNT1 inducible signaling pathway protein 1
NM_178751.2	−1,038	A730041O15Rik	RIKEN cDNA A730041O15 gene
NM_029688.2	−1,037	Npn3	neoplastic progression 3
NM_013692.1	−1,031	Tieg1	TGFB inducible early growth response 1
NM_013525.1	−1,025	Gas5	growth arrest specific 5
NM_175395.2	−1,023	1700001A24Rik; 6330578E17Rik	RIKEN cDNA 1700001A24 gene;RIKEN cDNA 6330578E17 gene
NM_019704.1	−1,023	C78915	expressed sequence C78915
NM_010234.2	−1,022	Fos	FBJ osteosarcoma oncogene
NM_011594.2	−1,020	Timp2	tissue inhibitor of metalloproteinase 2
NM_198420.1	−1,019	D15Wsu169e	DNA segment, Chr 15, Wayne State University 169, expressed
NM_008662.1	−1,018	Myo6	myosin VI
AK033214.1	−1,018	Gfm	G elongation factor
NM_010885.2	−1,013	Ndufa2	NADH dehydrogenase (ubiquinone) 1 alpha subcomplex, 2
NM_027287.1	−1,007	Dnajb4	DnaJ (Hsp40) homolog, subfamily B, member 4
NM_133942.1	−1,006	AA960558	expressed sequence AA960558
NM_007664.1	−1,005	Cdh2	cadherin 2
NM_027189.1	−1,004	Gemin7	gem (nuclear organelle) associated protein 7
NM_009627.1	−1,001	Adm	adrenomedullin

This Table represent a selection of annotated genes upregulated (red) and downregulated (green) by estradiol, sensitive to LEF-1 mutant. The list contains only those “annotated genes” with a log2R higher or lower than 1. **A** represent the control cell line (N2a-m-*mock transfected*). **B** represents the cell line transfected with *delta-56*-LEF-1. In the table **es**t, represents treatment with estradiol; and **Wnt**, treatment with Wnt3a.

We labeled two genes (***plasminogen*** **and** ***LEF-1***) regulated by estradiol or by Wnt3a , both were subsequently validated at protein level.

As shown in figure 9A, the log_2_ R of some of these genes was low, and certainly, the protein expression showed no differences. Considering that the low level of detection may be related with the short exposure time (60 minutes), we extended the treatment to confirm the differential regulation of some genes mediated by Δ56LEF-1. Accordingly, we treated the cell lines with estradiol or Wnt3a over a period of 24 h to allow for the accumulation of the protein. Soluble fraction, or nuclear fractions, or conditioned medium was obtained from treated cells and we analyzed the genes identified as differentially expressed in the microarray (when antibodies were available) as well as some genes known to be regulated by Wnt, such as E-cadherin, β-catenin or cyclin D.

Our data confirmed some of the variations in the genes described. Interestingly, MMP-2 secretion was most prominent in Wnt-treated cells whereas the proactive form appeared to be secreted prominently by Δ56LEF-1/N2a-m cells. Similarly, β-catenin displayed weaker protein expression in Δ56LEF-1/N2a-m cells, independently of the treatment.

Finally, a second set of genes were found, those that respond to estradiol (and/or Wnt) in control cells and whose regulation was modified by the presence of Δ56LEF-1. Indeed, longer treatment enabled clear differences to be detected at the protein level in genes such as N-cadherin and Cyclin D2. Our data indicated that N-cadherin was upregulated by estradiol and Wnt, and that the expression of Δ56LEF-1 almost completely blocked these effects (Figure 9B). While Cyclin D2 behaved similarly, Cyclin D1 appeared to be unchanged and unaffected by treatments (Figure 9B). On the other hand, E-cadherin was upregulated by estradiol in control cells (Group **A**), whereas the presence of Δ56LEF-1 generated an uncontrolled upregulation. This result revealed an important role for LEF-1 in estrogen-regulated E-cadherin expression.

## Discussion

Estrogens may act through different mechanisms, the best known of which involves their capacity to directly bind to and dimerize estrogen receptors (ERs). This hormone-protein complex can either bind directly to promoter estrogen response elements (EREs), or act as a cofactor at non-ERE sites through the interaction with other DNA-binding elements, such as AP-1, or c-Jun [5], [25]–[26]. A second mode of action is associated with the so-called “non genomic” or “rapid” actions, that include the activation of PKC, G-protein-coupled receptors, ERKs and PI3K/Akt in different cell systems [8], [27]. Regarding this theme, we recently reported that ERα forms a protein complex with GSK3 and β-catenin in the hippocampus of ovariectomized rats; the action of estradiol dissociated β-catenin from the complex in a manner that was clearly correlated with the inhibition of GSK3 [12]. The interaction of ERα with GSK3 does not appear to be specific for neurons and indeed, it has recently been demonstrated that ERα is also phosphorylated and modulated by GSK3 in other cell types [28].

### Estradiol, through a “rapid response”, activated the pathway Akt/GSK3, stabilized β-catenin, and modulates TCF-mediated transcription, in neurons

We had initially found that the inhibition of GSK3 by estradiol was correlated with the stabilization of β-catenin. Thus, our first aim here was to determine whether the pool of β-catenin regulated by estradiol acts as a co-transcriptional modulator using canonical TCF-mediated transcription or alternatively, whether the stabilization of β-catenin plays some other role. With this aim, we selected a neuronal cell line easily amenable to transfection with a TCF-reporter: the N2a-m cell line which expresses the α and β ER receptors and accumulates nuclear ER when treated with estradiol. Significantly, we detected a complex of β-catenin and GSK3 in these cells following exposure to either ERα or ERβ. When N2a-m or cortical neurons were treated with estradiol, we detected a similar biochemical response with an increase of GSK3 serine phosphorylation as well as the stabilization of β-catenin. This inhibition of GSK3 was time- and concentration-dependent, and these data are in essence similar to those described in the hippocampus of ovariectomiced rats after estradiol treatment, or in hippocampal neurons [12].

Taking into account these initial results, the question arose as to whether β-catenin stabilized by estradiol might modulate transcription. It is believed that β-catenin has two different and complementary roles in cells; it may contribute to the cell-cell adhesion or it may act as a co-transcriptional regulator of the TCF family [16]–[19]. Our data first showed that estradiol activated TCF-mediated transcription at concentrations of 10×10^−9^ M. This nuclear activation of *luciferase* was time- and concentration-dependent, it was maximal at 10–20×10^−8^ M, and it could be abolished by the ER antagonist ICI 182780. Similar, but slightly weaker transcriptional activation could be obtained with the ERα-selective agonist, PPT (5–10 nM), as well with the ERβ-selective agonist, DPN (5–10 nM). All these data strongly suggested that estradiol stabilizes β-catenin through ERα and ERβ, and that at least a portion of this protein pool can activate transcription. This transcriptional activation depended on the phosphorylation status of β-catenin. Indeed, the highest levels of transcription were obtained with a mutated form of β-catenin (S33Y) that was virtually unaffected by estradiol. These data raise the question as to whether this TCF- β-catenin mediated transcription is similar to that obtained with Wnt proteins.

We have also showed that estradiol may activate transcription from a portion of the *engrailed-1* promoter that contains well characterized TCF elements (pENP1-*luc*) (McGrew et al. 1999), and also activate transcription in primary neurons derived from mice transgenic for a TCF-β-galactosidase reporter driving the expression of a *luciferase* reporter. It is important to remember that the estradiol-dependent transcription mediated by pENP1-*luc* is severely inhibited by the expression a LEF-1 truncated-mutant (Δ56LEF-1) [23]. We found that estradiol slightly augmented the formation of DNA-TCF complexes, a similar effect to that produced by exposure to Wnt3a. This transcriptional activation is dependent on LEF-1, since antibodies against LEF-1 can disrupt DNA-complex formation. Furthermore, the presence of Δ56LEF-1 almost completely prevented estradiol-mediated transcription.

It was recently demonstrated that estradiol regulates LEF-1 and Tcf3, and more importantly, that a complex containing ERα and Tcf3/LEF-1 may be immunoprecipitated from mouse uterus extracts [29]. Accordingly, it was proposed that a complex composed of ERα/β-catenin/Tcf-3 is an important part of the estradiol response in this tissue. Although we cannot completely rule out this possibility (particularly since we did not use the same methodology), in our experiments LEF-1 was not immunoprecipitated when antibodies against either ERα or β were used. Only antibodies against LEF-1 prevented the formation of a DNA-protein complex in EMSA assays, unlike antibodies against Tcf 3 or ERα. Indeed, the initial cytoplasmic ERα/β-catenin/GSK3 complex appears to be different, with the complex in uterus being arranged (assembled?) after hormone addition, while in neural cells and neurons the complex is detected even prior to hormone treatment.

Further studies will be required to clarify whether the same ERα/β-catenin/Tcf-3 complex observed in the uterus is also present in neurons or in some specific brain regions. It will be interesting analyzing other possible components that might mediate the effect of the estrogen receptor on TCF/LEF-1 transcription, such as transducin beta-like protein 1 (TBL1) and its highly related family member TBLR1 [30]; among many others (see reviewed in [16]–[19]). A different point worthy of mention is that we detected multiple ERα bands in N2A cell extracts and after estradiol treatment only some of them appear to move into the nuclear fraction. This observation opens some interesting questions about the molecular nature of these multiple bands and why only some of them are mobilized. We are currently initiating the study of these ERα isoform/s in order to clarify the different possibilities suggested by the bibliography (isoform *versus* proteolysis??) [31]–[32].

Several publications have recently emerged in which new elements have been described that regulate the Wnt-β-catenin pathway. For example, c-Jun forms a complex with β-catenin and Dvl in the nucleus, and this association regulates the transcriptional activity of β-catenin [33]. Moreover, Rac1-JNK2 appears to act as a novel modulator of β-catenin mediated transcription [34]. Thus, the complex regulation of β-catenin is becoming more evident, as it is not only modulated through its cellular distribution or phosphorylation, but also by the proteins with which it associates. Here, we describe a novel pathway that regulates β-catenin/TCF activity through a ligand, estradiol, using at least some of the components of the original Wnt pathway.

### Is the gene expression through LEF-1/β-catenin estradiol-mediated similar to those triggered by Wnt?

To determine more specifically whether estradiol can regulate gene expression using the TCF/LEF-1-β-catenin system in a way similar to that of Wnt3a, we generated N2a-m cells stably transfected with Δ56LEF-1 cDNA. By analyzing the gene expression profile of these cell lines on an Aplied Biosystems Mouse Genome Survey Microarray, we detected 223 genes modified by estradiol; whereas Wnt3a modified 303 genes, sensitive to Δ56LEF-1 (see **Table 1**, containing only the annotated sequences).

To validate these changes, at the protein level, we had to extend the treatment period and as a result, we selected some representative genes from our array, such as *Plg* as well as some genes that have been previously associated with Wnt stimuli, such as E-Cadherin [35], Cyclin D [36]–[38], c-myc [39], LEF-1 [40]–[41] and TCF-1 [42]. We used commercially available antibodies to validate the changes in the levels of these proteins in the presence of Δ56LEF-1 and/or the hormone or Wnt. Our data revealed two major types of response: first, those genes modified initially by the LEF-1 mutant background, such as Plasminogen, LEF-1 , β-catenin and MMP-2, and second, a group of genes which initially responded to estradiol, either positively or negatively, and additionally showed an increased response in the Δ56LEF-1 cells, such as N-Cadherin, E-Cadherin and Cyclin D2. Indeed, the CyclinD2 promoter is clearly regulated by the estrogen receptor as well as by TCFs, although its responsiveness to Wnt proteins has also been assessed [36], [38]. Our data indicate that in neurons, this gene may be regulated by both elements, at least in part *via* β-catenin/LEF-1.

Taken together, these data demonstrate the existence of a new pathway controlled by estradiol, at least in neurons. Our data show that this pathway contains some elements that may belong to the canonical Wnt-pathway. Indeed some reports indicate that estradiol may control the response of the Wnt pathway as an important part of its neuroprotective role. This neuroprotective response is mediated by increase of Wnt3a and inhibition of Dkk1 elevation, triggered after cerebral ischemia [43]. More work is now required to identify whether a similar mechanism is specific for certain types of brain insults or represents a more general mechanism. The analysis of our arrays (**Table 1**) supports the hypothesis that only part of the estradiol response sensitive to LEF-1, is similar to Wnt3a response sensitive to LEF-1. This open the possibility that in addition of collaborate with the Wnt response, estradiol may regulated a pool genes (β-catenin/LEF-1- directed), independently of Wnt status.

### “Neuroprotective role” of estradiol and GSK3 inhibition

A complementary and important aspect of our data is that estradiol modulates the kinase activity of GSK3 irrespective of the neuronal source, and consequently the stabilization of β-catenin is an important event in these so called “non-genomic actions”. When considering the role of GSK3 and β-catenin in neurodegeneration [44], such as in relation to Alzheimer's disease or ischemia, some of the functional effects of estradiol may, at least in part, represent an important physiological control of this kinase [15]. We have to remember that in model systems of β-amyloid-mediated toxicity, similar concentrations of estradiol may protect neurons [15], [45], [46].

Estrogens play an important role in normal brain development [47], [48]. In addition, this hormone appears to exert a more general neuroprotective effect such as in mouse models of Parkinson's disease [49]–[53] or after brain ischemia [54]–[58]. In some ischemia models the estradiol–mediated GSK3 response, appears to be JNK-Dkk1-dependent [43], and we cannot discard this possibility in our neuronal system. However, our data indicate that the estradiol-mediated inhibition of GSK3 is PI3K-Akt dependent (O. Varea et al., unpublished data). It will be very important to determine whether the response that we observed is specific to “immature neurons” or whether it represents a more general mechanism.

## Materials and Methods

### Primary Cortical neurons and Neuroblastoma N2a-m

Mice were treated following the guidelines of Council of Europe Convention ETS123, recently revised as indicated in the Directive 86/609/EEC. In addition all protocols were approved by the institutional animal care and use committee. Cortical neurons were obtained from E18 mouse embryos after isolating the cortex in Ca^2+^- and Mg^2+^-free Hanks Buffer Salt Solution (HBSS 1×, GIBCO). Once 8–10 embryonic cortices were obtained, they were finely cited, washed twice in HBSS 1× buffer,and incubated in 0.25% trypsin (GIBCO) and 1 mg/ml DNAse (Roche) for 15 min at 37°C. Trypsin and DNAse were then eliminated by washing three times, with HBSS 1×, and the cut tissue was then triturated using a siliconized pipette. The cells were counted and plated in a poly-lysine coated (1 mg/ml, Sigma) 60 mm dish containing plating medium (MEM, 20% Glucose, horse serum and antibiotics). After 3 hours plating, the medium was changed to phenol red-free Neurobasal medium supplemented with B-B27 GIBCO) and the neurons were maintained under these conditions for 2 days. Murine neuroblastoma N2a-m cells were grown at 37°C in 7% CO_2_, in DMEM supplemented with 10% fetal calf serum (GIBCO) and 2 mM glutamine.

#### Cell treatments

One day before treatment, the medium was changed to serum and phenol red free-medium The compounds used for treatments were: 17-β-estradiol (stored in ethanol at a concentration of 1 mM), ICI 182780 (Tocris, stored in dimethyl sulfoxide -DMSO- at 2 mM), Wnt3a recombinant protein (R&D systems, dissolved in PBS-BSA 0.1% and stored at 0.1 µg/µl), and the estrogen receptor agonists, 4,4′,4″-(propyl-[(1)H]-pyrazole-1,3,5-triyl) trisphenol (PPT, α-selective) and 2,3-bis (4-hydroxyphenyl) propionitrile (DPN (β-selective) agonists, both dissolved in ethanol. Different concentrations of these compounds were used as indicated for each experiment and the controls received the vehicle of each compound alone, ethanol in the case of estradiol, DMSO for ICI 182780 and PBS-BSA 0.1% as control for Wnt3a. The final concentration of ICI 182780 used was depending on the concentration of estradiol, in a ICI/estradiol ratio of 100 times.

### Western Blotting and Antibodies

After two washes with PBS, cell extracts were prepared in lysis buffer containing 200 mM Hepes pH 7.4, 100 mM NaCl, 100 mM NaF, 1 mM Na_3_VO_4_, 5 mM EDTA, 1% Triton and a protease inhibitor cocktail (Roche). The cells were left for 30 min on ice in this lysis buffer and then collected with a cell scraper. After adding loading buffer, the samples were boiled for 10 min and resolved by Tris/Glycine SDS-Polyacrylamide gel electrophoresis, and the proteins were then transferred to a nitrocellulose membrane (Amersham) in the presence of 20% methanol and 0.1% SDS. Non-specific signals were blocked by incubating the membrane in PBS-Tween-20 (PBT) and 5% milk for 2 h. The antibodies used to probe the membranes were raised against: Ct ERα 1∶800 (MC-20, Santa Cruz), ERβ 1∶1000 (H-150, Santa Cruz), GSK3 α/β 1∶1000 (Cell signalling), GSKPSer^9, 21^ 1∶1000 (Cell Signalling), β-catenin 1∶800 (Transduction Labs), non-phospho β-catenin 1∶800 (Upstate), actin 1∶2000 (Sigma), LEF-1 HMG box 1∶800 (Sigma), cyclin D1 1∶1000 (Santa Cruz), cyclin D2 1∶1000 (MBL), N cadherin 1∶1000 (Sigma), E cadherin 1∶800 (Santa Cruz), MMP2 1∶200 (Santa Cruz), nucleolin 1∶1000 (Santa Cruz), beta-galactosidase (ICN Biomed-Cappel), β-III tubulin (Chemicon). These antibodies were then detected with horseradish peroxidase conjugated secondary antibodies (Amersham) used at a dilution of 1∶5000.

### Immunocytochemistry

To study the localization of proteins by immunocytochemistry, we fixed cells plated at low density with 4% paraformaldehyde for 20 min at RT. After 3 washes for 10 min in PBS, fixed-cells were incubated with 50 mM NH_4_Cl and then blocked and permeabilized for 30 min with 1% fetal calf serum and 0.1% Triton-X-100 in PBS. The cells were incubated with the primary antibodies for 2 h at RT and after 3 washes with blocking solution, the secondary Alexa fluor-488 or 594 antibodies were added (1∶1000, Molecular Probes). The primary antibodies used were raised against β-catenin (1∶400, Transduction Labs) or ERα (1∶500, MC-20, Santa Cruz). To visualize the distribution of polymerized actin, fixed cells were stained with phalloidin conjugated to Alexa 488 or 594, as indicated in each experiment (1∶100, Molecular Probes). Coverslips were mounted with Fluoromount G (Southern Biotechnology Associates, Inc) and photographs were taken on a confocal microscope (LSM 510 META, Zeiss).

### Plasmids and Transient expression in N2a-m cells

The plasmids used for transient expression were: pTOPFlash (Upstate), containing three copies of the consensus sequence recognized by the TCF/LEF transcription factors (CCTTTGATC) and with the c-Fos promoter driving the expression of luciferase gene; pFOPFlash (Upstate) that contains three non-functional copies of the TCF/LEF binding site (CCTTTGGCC); and EGFP-N1 encodes an enhanced green-fluorescent protein used to quantify the efficiency of transfection when co-transfected with TOP/FOPFlash. N2a-m cells were transfected using LipofectAMINE 2000 (Invitrogen) according to the manufacturer's instructions. For transfection, 600,000 cells were plated in a 60 mm-culture dish with growth medium and the day before transfection, the medium was changed to DMEM without FCS or antibiotics. Co-transfections were performed with 2 µl LipofectAMINE (Invitrogen) and 1 µg of total-DNA (0.75 µg TOP/FOP plus 0.25 µg EGFP-N1). After transfection, the medium was changed to DMEM without phenol red or FCS, supplemented with an antibiotic cocktail. Treatments were started 48 h after transfection to coincide with the maximum expression levels of the co-transfected proteins.

Other plasmids used for transfection were: empty pcDNA3 as an internal control; Δ45 β-catenin, a deleted form of β-catenin lacking the N-terminal (kindly provided by Dr. R. Moon); and LEF-1Δ56 (kindly provided by Dr. R. Grosschedl) that expresses a form of LEF-1 that is unable to interact with β-catenin.

### Primary Cortical Neuron Nucleofection

The TOPFlash plasmid was introduced into cortical neurons by nucleofection (Basic Nucleofector Kit for Primary Mammalian Neural cells, Amaxa Bioscience) according to the manufacturer instructions. A total of 3 µg TOPFlash plus 1 µg GFP was introduced into 4×10^6^ cells, and both luciferase activity and the efficiency of nucleofection were analyzed after 2 days in culture.

### Assay of luciferase activity

After treatment of the TOPFlash transfected N2a-m cells, they were incubated in 60 mm plate with 1 ml of TEN buffer (40 mM Tris-HCl pH 7.5, 10 mM EDTA, 150 mM NaCl) for 15 min at RT to detach the cells from the dish. Once the cells had been collected and pelleted, they were resuspended in 100 µl of lysis-buffer (100 mM Potassium Phosphate buffer ph 7.8, 1 mM DTT, 0.5% Triton) and incubated on ice for 30 min. The lysate was then centrifuged at 4°C, 5 min to eliminate aggregates and the supernatant was transferred to a fresh tube. A fraction of this supernatant was resolved by electrophoresis to check the cellular mass and the amount of protein was quantified. The rest of the cell extract was used to measure the luciferase activity in 200 µl of luciferase buffer (25 mM Glycyl-glycine, 15 mM MgSO_4_, 5 mM ATP, 1 mM DTT, 100 µg/ml BSA) mixed with 20 µl of the cell extract. The luciferine reaction substrate (Promega) was used at a concentration of 1 mM (dissolved in sterile water), and we determined the quantity of luciferase expressed in the cells by measuring the reaction at 560 nm with a luminometer (Monolight® 2010, Analytical Luminescence Laboratory). The values obtained correspond to the mean and standard deviation of three sets of independent experiments. To analyze the induction of β-catenin co-activator function, the values were normalized to controls (corresponding to the ratio transfected/non treated cells).

### Immunoprecipitation

Inmunoprecipitation assays we carried out on a confluent culture dish of N2a-m cells that were collected in 0.5 ml of immunoprecipitation buffer A (1% Triton X-100, 150 mM NaCl, 10 mM Tris pH 7.4, 1 mM EDTA pH 8, 1 mM EGTA pH 8, 0.2 mM sodium-*ortho*-vanadate, 0.2 mM PMSF, 0,5% NP-40). After incubation on ice for 30 min, the lysate was centrifuged (16000×g at 4°C, 15 min) to eliminate possible aggregates. The supernatant (100 µl of total native lysate) was incubated in the following buffer: 2% Triton X-100, 20 mM Tris pH 7.4, 2 mM EDTA pH 8, 2 mM EGTA pH 8, 0.4 mM sodium *ortho*-vanadate, 0.4 mM PMSF, 1% NP-40) in the presence of 5 µg of anti-ERα antibody in a total volume of 500 µl. The mixture was incubated for 1 h at 4°C and subsequently, 10 ul of Protein A-Agarose solution (Sigma) was added and incubated at 4°C for 30 min with agitation. The agarose beads were recovered by centrifuging at 16000×g for 4 min at 4°C, and the pellet was washed three times with buffer A and centrifuged (16000×g at 4°C). Finally, the pellet was resuspended in 30 µl of 2× electrophoresis sample buffer (250 mM Tris pH 6.8, 4% SDS, 10% glycerol, 0.006% bromophenol blue, 2% β-mercaptoethanol), boiled for 10 min and loaded onto an SDS-PAGE gel to be analyzed by western blotting.

### Purification of cortical nuclei

After the dissection of the cortex from 12–16 E18 embryos, the tissue was homogenized in buffer A (0.32 M sucrose, 10 mM Tris pH 7.4, 3 mM MgCl_2_ supplemented with 0.1% Triton-X-100, protease inhibitors, DTT and PMSF). The homogenate was centrifuged at 2500 rpm for 10 min at 4°C, the supernatant was discarded and the pellet was resuspended in buffer A without Triton, and centrifuged in the same conditions. The pellet obtained was resuspended in buffer B (the same as buffer A, but containing 1.9 M sucrose instead of 0.32 M) and homogenized again. The extract was carefully loaded over buffer C (buffer A, but with 2 M sucrose) and spun at 12000 rpm for 60 min at 4°C. The final pellet of the nuclei was resuspended in 50 µl of buffer A, 150 µl of protein extraction buffer (20 mM Tris pH 7.4, 400 mM NaCl, 0.5 mM EDTA pH 8, 0.5 mM EGTA pH 8, 2 mM MgCl_2_) was added and the mix was incubated on ice for 30 min. To finally obtain the soluble fraction of nuclear proteins, the extract was centrifuged at 35000 rpm for 30 min at 4°C and the protein concentration in the extracts was measure using the Lowry reaction.

### Electrophoretic Mobility Shift Assays (EMSA)

To obtain nuclear extracts of N2a-m or cortical neurons, cells were grown in serum free-DMEM, without phenol red, to 80% confluence. After washing twice with PBS, 400 µl of EMSA buffer A was added to each P100 dish (10 mM Tris pH 7.5, 10 mM KCl, 0.1 mM EDTA, 0.1 mM EGTA,1 mM DTT, PMSF, 1 µM Na_3_VO_4_ and protease inhibitor cocktail). Once two confluent P100 mm dishes were collected in a fresh tube (8 P60 mm in the case of cortical primary neurons), cells were incubated for 15 min on ice and then 40 µl of 10% NP-40 was added and vortexed for 20 sec. The supernatant was centrifuged for 30 sec at 15000 rpm to obtain the cytoplasm fraction. Once dry-resuspended the pellet containing the nuclei, 50 µl of EMSA buffer B (20 mM Tris pH 7.5, 400 mM NaCl, 0.5 mM EDTA, 0.5 mM EGTA, 2 mM MgCl_2_, 1 mM DTT, 0.5 mM PMSF, 1 µM Na_3_VO_4_ and protease inhibitor cocktail) was added at 4°C with vigorous shaking for 15 min. The samples were then centrifuged for 30 sec at 15000 rpm and the supernatant recovered as the nuclear extract. The EMSA assay was performed as described previously [59]. Protein-primer binding was carried out with 20 µg of nuclear proteins during 30 min at RT and a similar amount of each primer (50.000 cpm/µl annealed primer) was added to the reaction at a concentration of 1 mg/ml (sequence forward: 5′-GTCGCCCTTTGATCTTACC-3′, reverse: 5′-GTCGGGTAAGATCAAAGGG-3′, synthetic oligonucleotides from Invitrogen). When antibodies were used in the EMSA assays, 2 µg of antibody against TCF-1-X, TCF-3-X and LEF-1-X (from Santa Cruz) and 1 µg of antibody against β-catenin (Transduction Labs) or 0.8 µg of antibody against ERα (MC-20, Santa Cruz) was added 20 min after the binding reaction had terminated. Competition experiments were performed to recognize the specific bands corresponding to the TCF/LEF-1-DNA complex using TCF/LEF containing the mutated sequence (sequence forward: 5′-GTCGCCCTTTGGCCTTACC-3′, reverse: 5′-GTCGGGTAAGGCCAAAGGG-3′) and unlabelled-wild type TCF/LEF sequence at different final concentrations (20 ng and 100 ng). In all cases, the binding reaction was carried out at a final concentration of 60–65 mM KCl and in the presence of poly dIdC (1 mg/ml) and CHAPS (20%). The results of the binding reaction were analyzed in 4.5% acrylamide/bisacrylamide gels run at constant 130 V and dried (80°C, 1 h).

### Total RNA isolation and RT-PCR quantification

Total RNA was isolated from N2a-m cells from a confluent P100 mm dish or from 3 P60 mm dishes in the case of cortical neurons. An additional set of RNA was obtained from N2a-m stable-transfected with pcDNA3-LEF-1 56 or with empty-pcDNA3, either untreated or exposed to estradiol or Wnt3a. RNA was obtained using TRIzol (GIBCO) and it was finally resuspended in sterile-DEPC treated water. The RNA concentration was determined by spectrophotometry at 260 nm, and its integrity was checked using a Bioanalyzer Chip (Agilent). First strand cDNA synthesis was performed on 2 µg of RNA and using the reverse primer as the priming site, hybridized at 37–40°C during 5 min before the remaining reaction components were added: MMLV Reverse-Transcriptase, 2.5 mM DTT, and RT-Buffer (GIBCO) with RNAsin and 10 mM dNTPs. Elongation was performed at 37°C (optimal temperature for the RT enzyme) and the reaction was finally heated to 94°C (5 min) to inactivate the RT enzyme. RT-PCR amplification was performed to analyze cyclin D1, c-myc, actin, β-galactosidase, actin and GADPH expression using the Master SYBR Green I mix (Roche) with 2 mM MgCl_2_ in a lightcycler instrument (Roche Molecular Biochemicals). PCR was performed under optimal conditions for each primer pair. The oligonucleotide sequences and PCR conditions used were as follows: **actin**: 94°C-15 s, 57°C-10 s, 72°C-20 s (forward: 5′-TGTTTGAGACCTTCAACACC-3′; reverse: 5′-TAGGAGCCAGAGCAGTAATC-3′; 600 bp), **cyclin D1:** 94°C-15 s, 55°C-10 s, 72°C-20 s (forward: 5′-CACAACGCACTTTCTTTCCA-3′; reverse: 5′-GACCAGCCTCTTCCTCCAC-3′; 164 bp), **β-galactosidase:** 94°C-15 s, 64°C-5 s, 72°C-15 s (forward: 5′-ATCCTCTGCATGGTCAGGTC-3′, reverse: 5′-CGTGGCCTGATTCATTCC-3′; 315 bp).

### Transgenic mice

The transgenic mice containing the lacZ gene under the control of three consensus TCF/LEF-binding motifs upstream of the c-fos promoter have been described previously [21] (strain Tg(Fos-LacZ)34Efu/J, JAX®Mice, The Jackson Laboratory) . The genotype of the mice was checked using the following primers and PCR conditions: lacZ rose gene (forward: 5′-ATCCTCTGCATGGTCAGGTC-3′, reverse: 5′-CGTGGCCTGATTCATTCC-3′, 315 bp), endogenous gene (forward: 5′-CAAATGTTGCTTGTCTGGTG-3′; reverse: 5′-GTCAGTCGAGTGCACAGTTT-3′, 210 bp). The mouse colony was maintained in heterozygosis and it was necessary to check each embryo when used for primary neuronal cultures. Accordingly, a portion of the cortex was placed in a mixture containing “Dye Solution” (5 mM K_4_[Fe(CN)_6_].3H_2_0, 5 mM K_3_[Fe(CN)_6_], 2 mM MgCl_2_) and X-Gal (1 mg/ml).

### Microarray analysis

Gene expression profiles were generated using Applied Biosystems Mouse Genome Survey Microarray. Total RNA pools were cleaned using the RNeasy kit from Qiagen (Hilden, Germany). Digoxigenin-UTP labeled cDNA probes were generated and linearly amplified from 1 µg of each RNA pool using Applied Biosystems Chemiluminescent RT-IVT labeling Kit. Microarray hybridization, chemiluminescence detection, image acquisition and analysis were done according to Applied Biosystems protocols and the 1700 Chemiluminescent Microarray Analyzer.

Applied Biosystems Expression System software was used to extract Assay Signal, and Assay Signal to Noise ratio values from the microarray images. Bad spots flagged by the software were removed from the analysis. The Assay Signal of the represented genes was ***log*** transformed, loess-normalized (http://www.bioconductor.org) and further filtered by standard Expression Array System Signal to Noise threshold (S/N greater than 3 in at least one sample). The expression profiles of the two control populations (N2a-m stable-cell lines with or without ΔLEF-1) were compared with the corresponding population, treated with either estradiol or Wnt3a.

## Supporting Information

Figure S1 N2a-m cells express both the ERα and ER{lower caes beta} isoforms of the estrogen receptor and they respond to estradiol. Immunocytochemistry with anti-ERα and ERβ identified a different cytoplasmic location for the two isoforms in both cell lines. While ERα presented was diffusely distributed throughout the cytosol and nucleus, and it accumulated in the nucleus after the addition of estradiol (A–B). ERβ distribution was more heterogeneous and it could be found in aggregates, diffusely distributed in the cytosol or in the nucleus(C–D). The addition of estradiol (B and D) increases the immunoreactivity against two receptors in the nucleus. Images were analyzed by confocal microscopy and western blotting confirmed the expression of both isoforms in total cell extracts from control cells.
(9.68 MB EPS)

Figure S2 Distribution of β-catenin after exposure of N2a-m cells to estradiol does not reveal the translocation of β-catenin to the nucleus. Cell fractions corresponding to the soluble, membrane and nuclear fractions were analyzed in control and estradiol treated cells. Neither 30 nor 60 min exposure to estradiol provoked the nuclear accumulation of β-catenin, statistically significant. In contrast, recruitment of β-catenin to the membrane fraction was more evident. We also checked the movement of ERα as a control of the assay, which clearly accumulated in the nuclear fraction (see Figure 8).
(2.36 MB EPS)

Figure S3 N2a-m cells are responsive to Wnt3a protein. (A)- N2a-m cells respond to Wnt3a (20 ng/ml) with the accumulation of β-catenin and with a maximal effect observed after 90–120 minutes. (B)- The transcriptional activity of β-catenin was analyzed with the TCF-luc reporter. Luciferase activity was calculated at different concentrations of Wnt3a protein, as indicated above, and Wnt3a produced a significant increase in β-catenin/TCF dependent transcription. Asterisk represents P value from Student's t-test: * (P≤0.05), when compared the two Wnt concentrations; and ** (P≤0.01) when compared with control.
(1.61 MB EPS)

Figure S4 Neither ERα nor TCF-3 antibodies modify the migration of nuclear protein extracts from N2a-m cells exposed to estradiol or Wnt3a. Nuclear protein extracts were obtained from control or estradiol/Wnt3a treated cells as indicated in each lane. The incubation with specific antibodies against ERα or TCF3 did not produce a higher molecular weight band that migrated more slowly than that previously identified as a TCF-DNA complex, nor did they prevent the formation of the complex.
(2.50 MB EPS)
